# Supplementary material for: Mapping anthropogenic mineral generation in China and its implications for a circular economy
Source: Nat Commun. 2020 Mar 25;11:1544. doi: 10.1038/s41467-020-15246-4 (PMC7096490; doi:10.1038/s41467-020-15246-4)
Supplement: Supplementary file 1 — Supplementary Information [file 41467_2020_15246_MOESM1_ESM.pdf]

Mapping anthropogenic mineral generation in China and its implications for a circular economy

Zeng et al.

Supplementary Information

Contents

Supplementary Fig. 1 Relationship among the production, consumption, and theoretical consumption S1
Supplementary Fig. 2 Evolution of "Four Big Items" from 1970s to 2000s S2
Supplementary Fig. 3 The concise diagram of research procedure for this study S3
Supplementary Fig. 4 Classification of EEE, vehicle, and wiring & cable in China S4
Supplementary Fig. 5 Sensitivity analysis and uncertainty analysis with 95% confidence intervals S5
Supplementary Fig. 6 Probability density function of Weibull distribution S6
Supplementary Fig. 7 Data regression of net production of EEEs for P(x) S7
Supplementary Fig. 8 Data regression of net production of vehicle for $P(x)$ S9
Supplementary Fig. 9 Linear regression of imported scraps for I'(x) S13
Supplementary Fig. 10 Estimation of China's AM from 2010 to 2050: Range for $D(x)$ (Mt) S14
Supplementary Fig. 11 Resources weight in yearly-generated three AMs for $D_{m,j}$ S17
Supplementary Fig. 12 Relative economic shares of materials in total AM S18
Supplementary Fig. 13 The comparison of typical AM estimation S19
Supplementary Fig. 14 Validation for all the registered vehicles quantity S20
Supplementary Table 1 $P(x)$: Statistics of domestic production for EEE, vehicle, and wiring & cable in China S21
Supplementary Table 2 $I(x)$ and $E(x)$: Statistics of importation and exportation in China S24
Supplementary Table 3 w_i: Weights of each product and those data distributions used for Monte Carlo simulation S26
Supplementary Table 4 c_{ij}: Average content of resources contained in various products S27
Supplementary Table 5 Resource market prices in recent years (US$/ton) S31
Supplementary Table 6 Previous applications of various methods for predicting AM generation S32
Supplementary Table 7 Data demand's comparison of various methods for AM prediction [67] ... S32
Supplementary Table 8 w_i: Estimation of average PC weight 33
Supplementary Table 9 w_i: Estimation of average TV weight S33
Supplementary Table 10 Prohibitive imported directory of solid waste and their implemented time S34
Supplementary Table 11 $I'(x)$: Estimation and range of imported AM in 2010-2050 .... S39
Supplementary Table 12 η, β, and L: Parameters of Weibull lifespan distribution and the regulated maximum lifetime S42
Supplementary Table 13 $f(x)$: Lifetime distribution function of all the relevant products S43
Supplementary Table 14 The total estimated consumption of various product from 1990 to 2050 .... S46
Supplementary Note 1: Terminologies definition and boundary S54
Supplementary Note 2: The abbreviations and acronyms of main vocabulary S55
Supplementary Note 3: Available data pre-mining for estimation S56
Supplementary Note 4: Comparison of this study to other studies and real-world data S56
Supplementary References S57

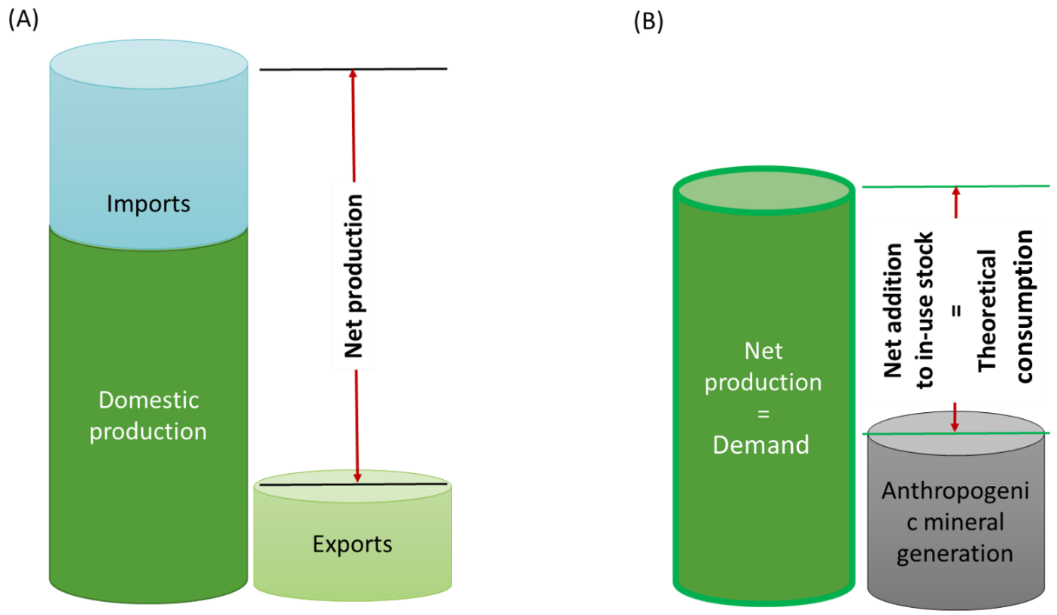

Supplementary Fig. 1 Relationship among the production, consumption, and theoretical consumption

Supplementary Fig. 2 Evolution of “Four Big Items” from 1970s to 2000s

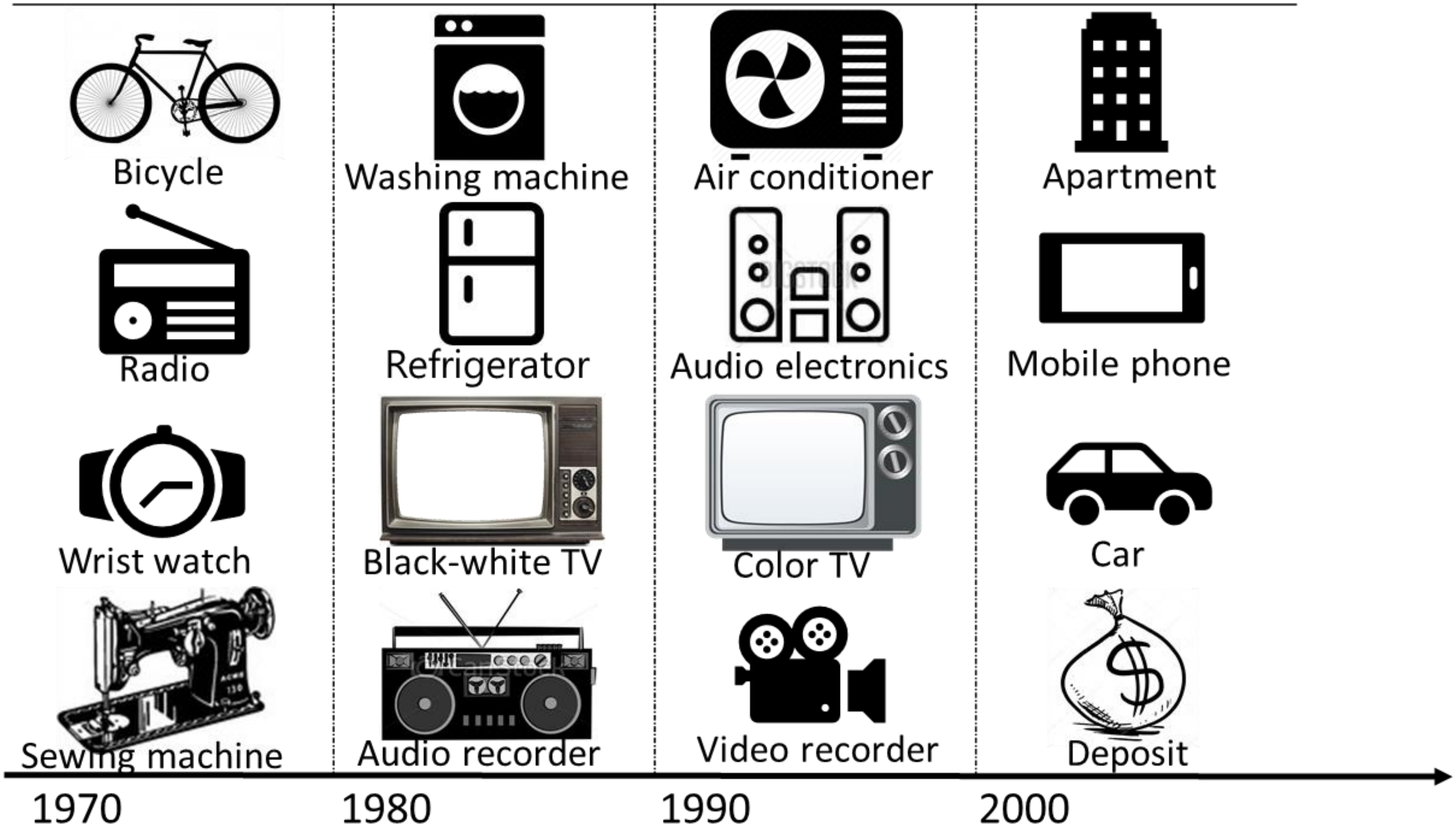

Note: The “Four Big Items” is a term originally applied to the four symbols of material success in China few decades years ago, especially while the young man has a marriage. Source from refes[1, 2, 3, 4].

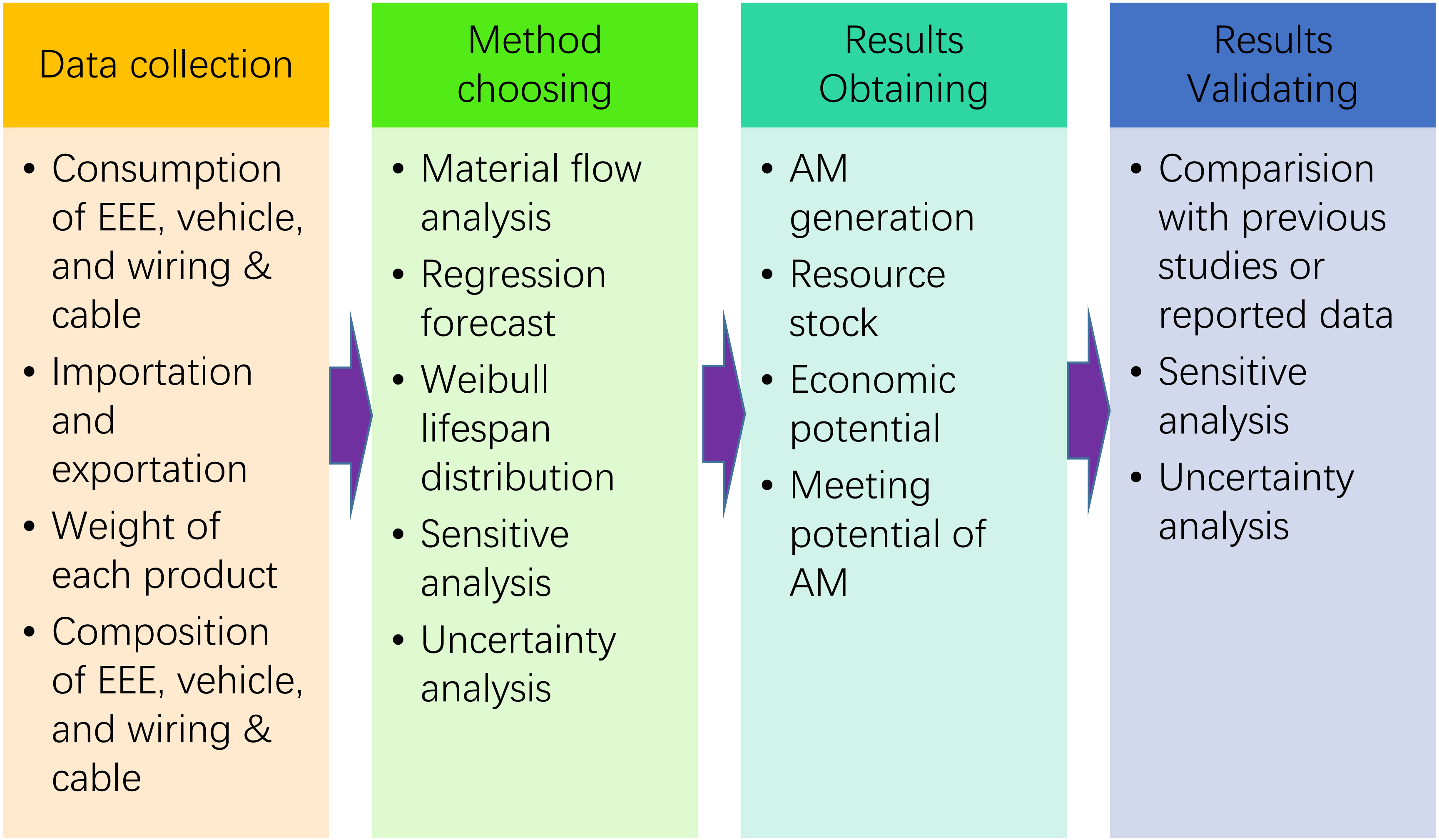

Supplementary Fig. 3 The concise diagram of research procedure for this study

Note: The choose of sensitive analysis and uncertainty analysis is relied upon the integrity of data. If only knowing the error or range of data, sensitive analysis will be used; if knowing not only the error or range, but the distribution of data, uncertainty analysis could be adopted.

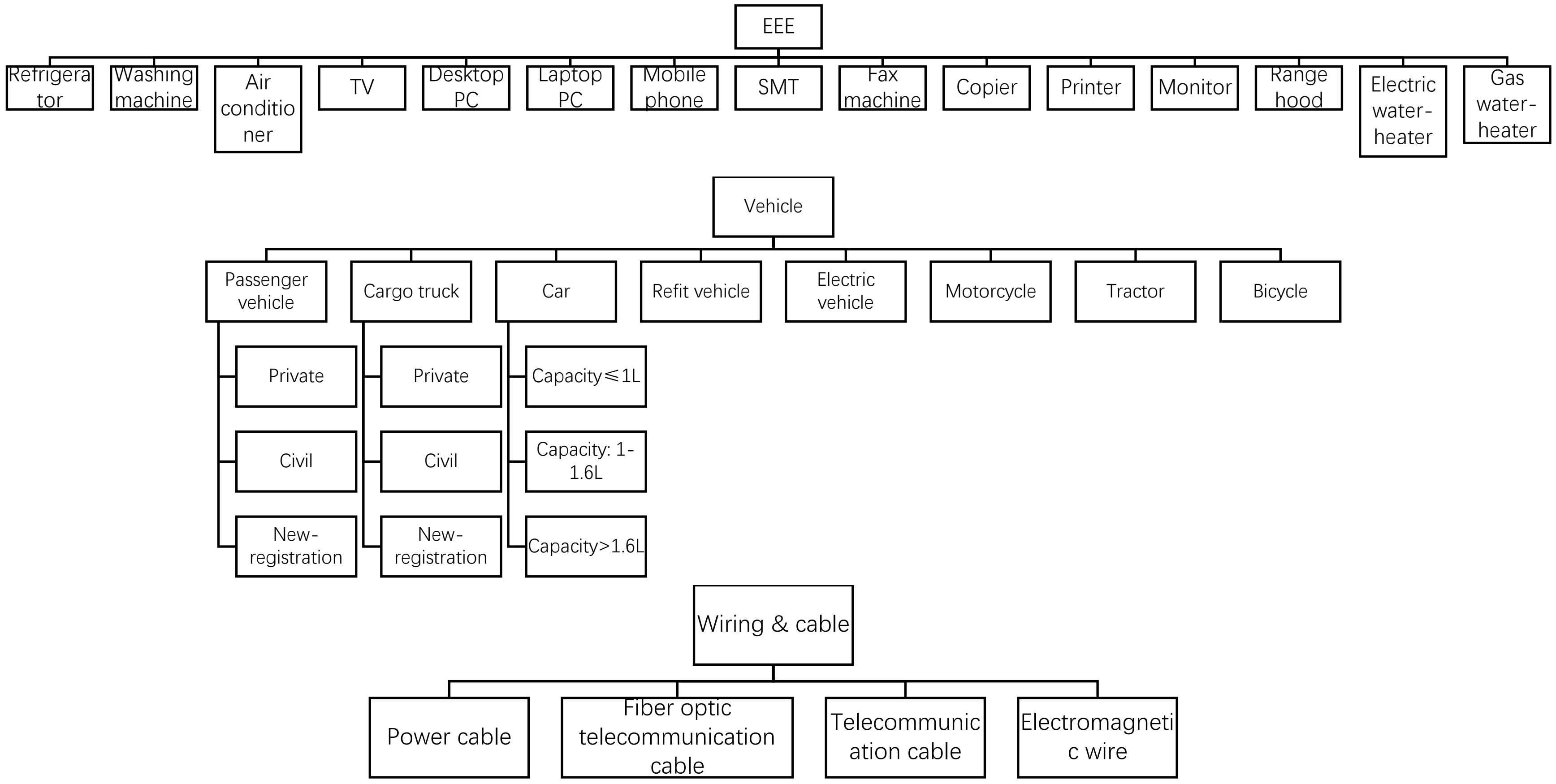

Supplementary Fig. 4 Classification of EEE, vehicle, and wiring & cable in China

Note: EEE, electrical and electronic equipment; TV, television; PC, personal computer; SMT, single-machine telephone.

Supplementary Fig. 5 Sensitivity analysis and uncertainty analysis with 95% confidence intervals

a share of importation on total AM, obtained from the indefinite importation dividing total AM. **b** total weight of AM in 2017 affected by each weight and parameters. **c** Cu weight of yearly-generated AM in 2017 affected by resources content and each WEEE weight. **d** total weight of AM in 2030 affected by each weight and parameters. **e** Al weight of yearly-generated AM in 2030 affected by resources content and each WEEE weight. Note: Min, Average, and Max indicate the minimum importation, average (or estimation) importation, and maximum importation, respectively. Monte Carlo simulation (10^5 iterations) was used to perform the uncertainty analysis with the Oracle Crystal Ball software.

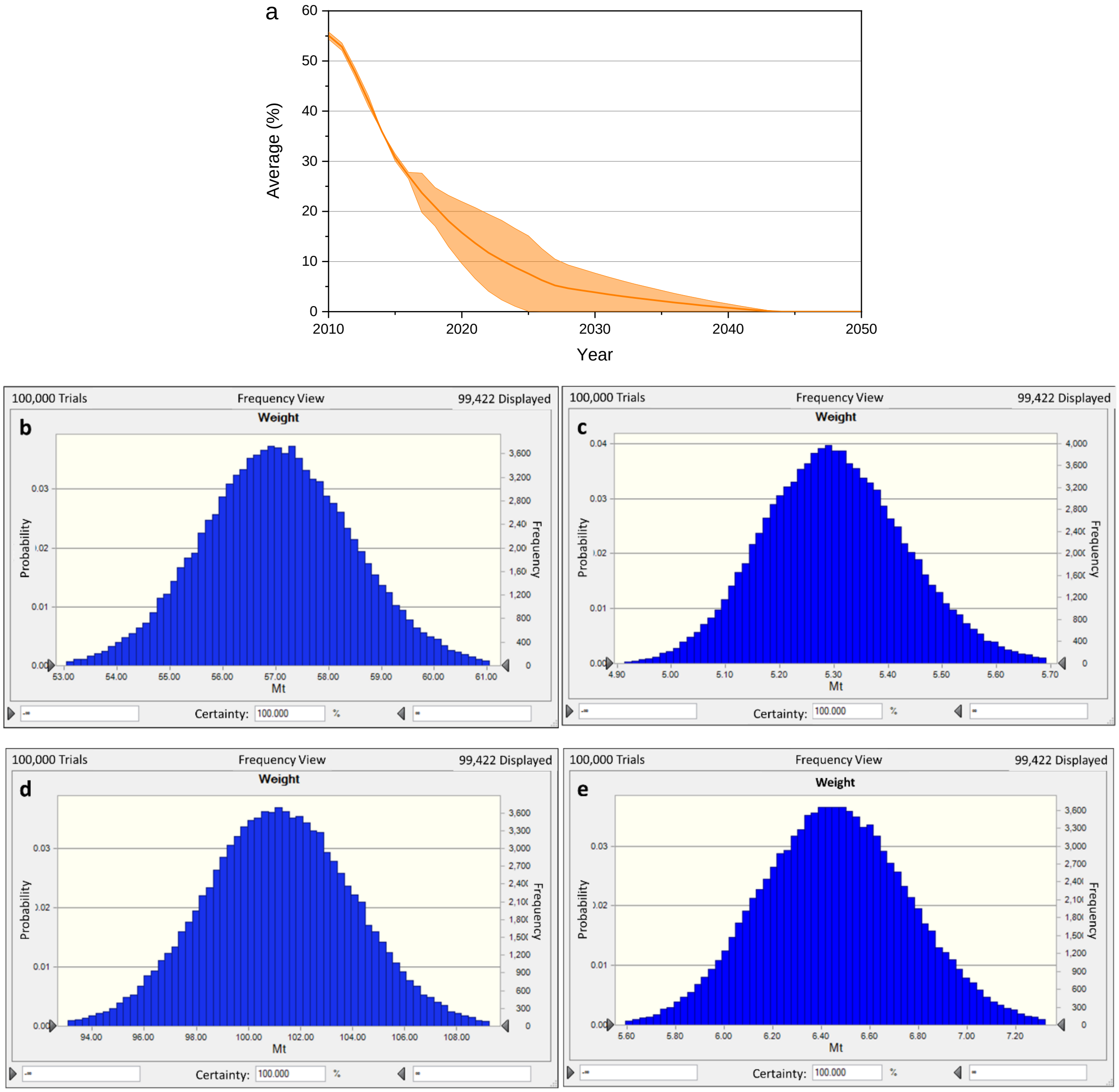

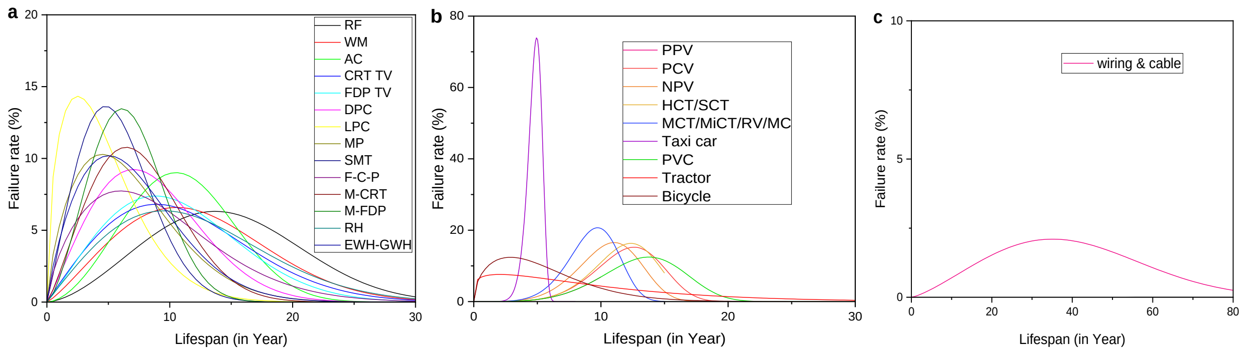

Supplementary Fig. 6 Probability density function of Weibull distribution

a EEEs[5]; **b** vehicle & bicycle; **c** wiring & cable. Note: RF: refrigerator; WM: washing machine; AC: air conditioner; CRT-TV: TV with cathode-ray tube (CRT); FDP-TV: flat display panel TV; DPC: desktop personal computer; LPC: laptop personal computer; MP: mobile phone; SMT: single-machine telephone; F-C-P: fax machine, copier, and printer; M-CRT: CRT monitor used for mainframe; M-FDP: FDP monitor used for mainframe; RH: range hood; EWH: electric water heater; GWH: gas water heater; PPV: Private passenger vehicle; PCV: Passenger civil vehicle; NPV: New-registration passenger vehicle; HCT: heavy cargo truck; SCT: small cargo truck; MCT: medium cargo truck; MiCT: mini cargo truck; RV: refit vehicle; MC: motorcycle; PVC: Private vehicle for civil purpose.

Supplementary Fig. 7 Data regression of net production of EEEs for *P*(x)

a RF; **b** WM; **c** AC; **d** TV; **e** DPC; **f** MP; **g** RH; **h** LPC; **i** Monitor; **j** Copier; **k** Printer; **l** EWH; **m** GWH; **n** SMT.

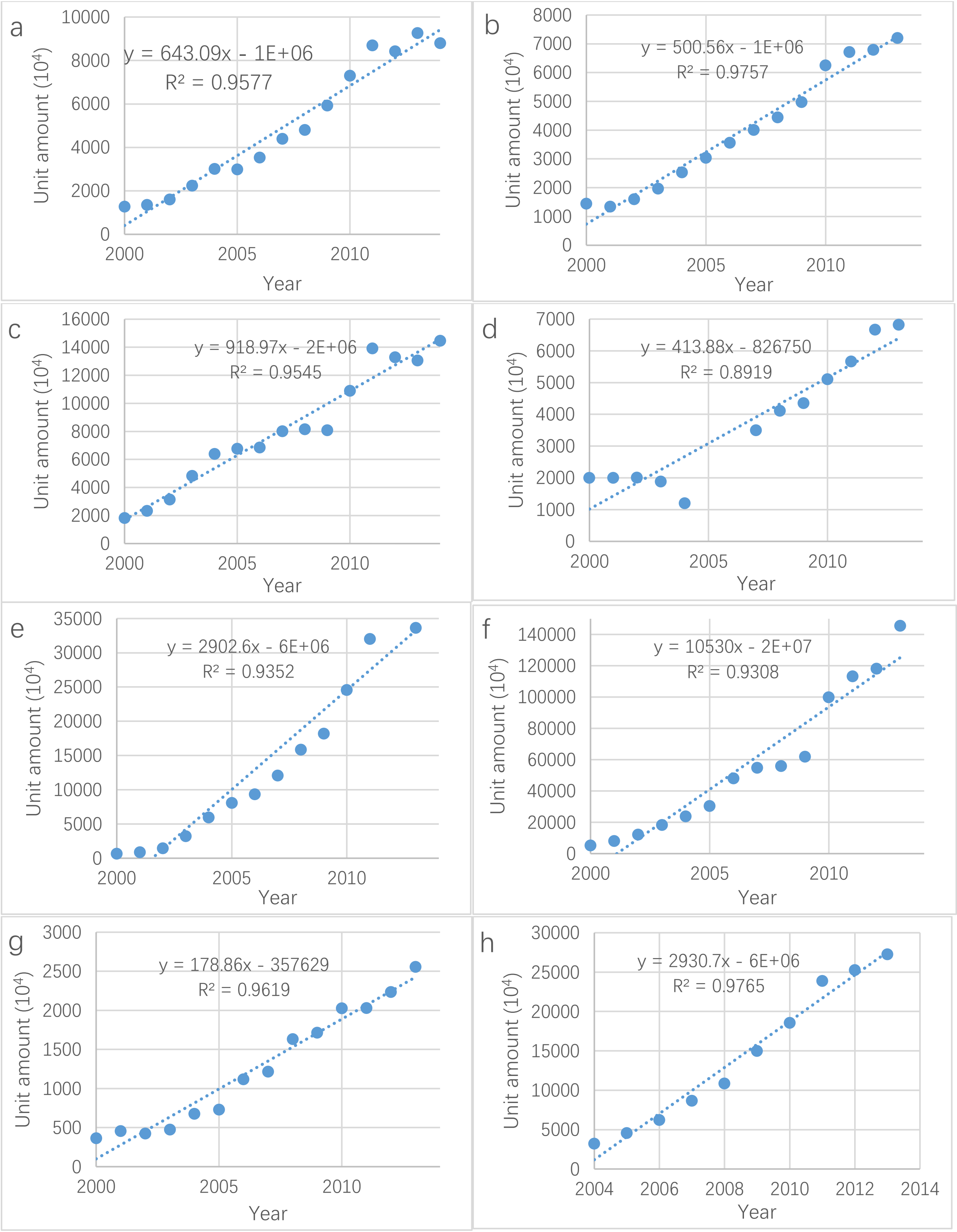

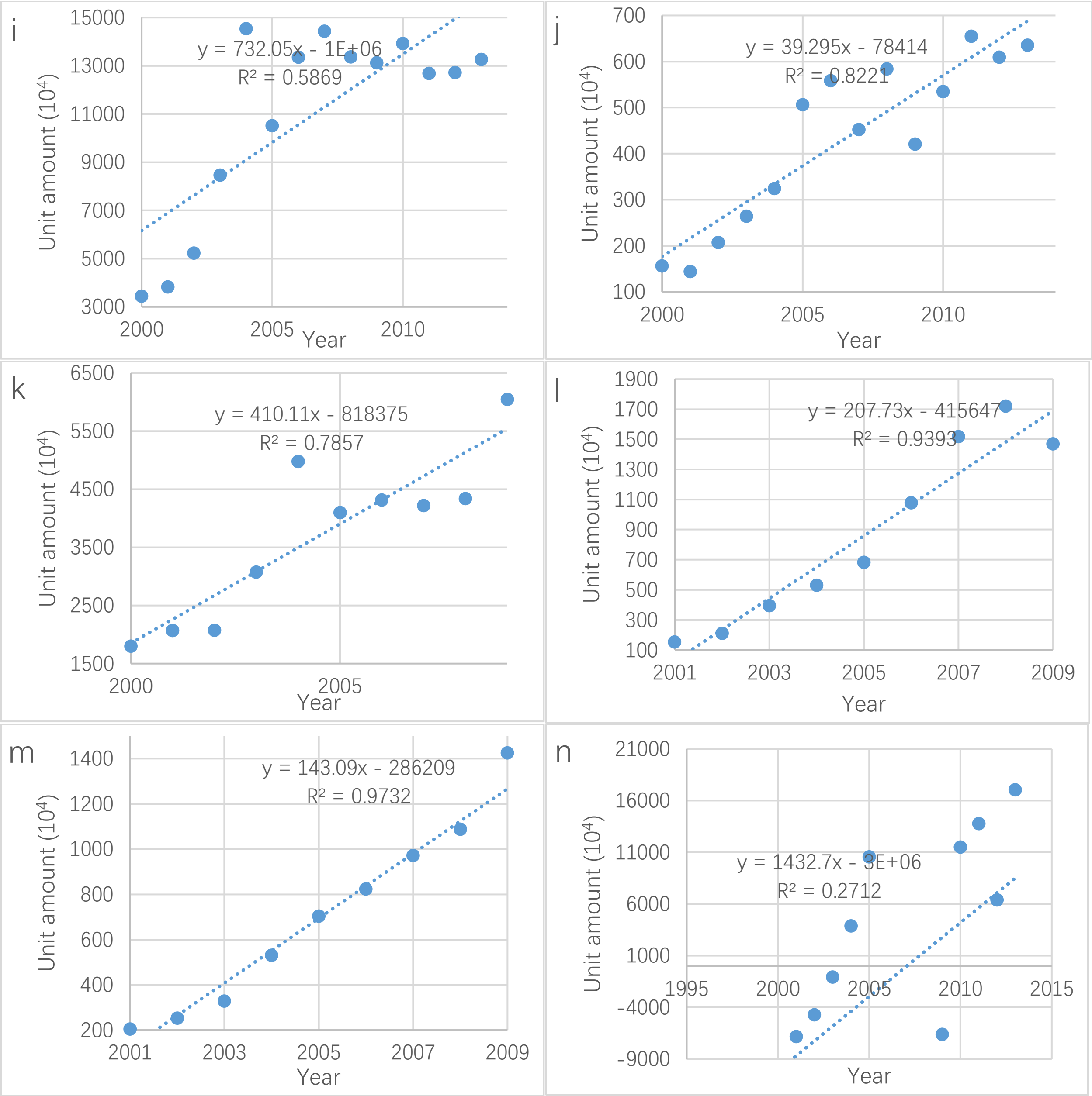

Note: rapid growth occurs at the start stage of the "inverted-U" shape.

Supplementary Fig. 8 Data regression of net production of vehicle for $P(x)$

a Small passenger vehicle; **b** Mini passenger vehicle; **c** New-registration civil & large passenger vehicle; **d** New-registration civil & small passenger vehicle; **e** Civil & large passenger vehicle; **f** Civil & small passenger vehicle; **g** Civil & mini passenger vehicle; **h** Heavy & private cargo truck; **i** Medium & private cargo truck; **j** Small & private cargo truck; **k** Mini & private cargo truck; **l** Heavy & new-registration cargo truck; **m** Small & new-registration cargo truck; **n** Mini & new-registration cargo truck; **o** Heavy & civil cargo truck; **p** Small & civil cargo truck; **q** Mini & civil cargo truck; **r** Car with capacity≤1L; **s** Car with capacity of 1-1.6L; **t** Car with capacity>1.6L; **u** Refit vehicle; **v** Motorcycle; **w** Tractor; **x** Bicycle; **y** EV.

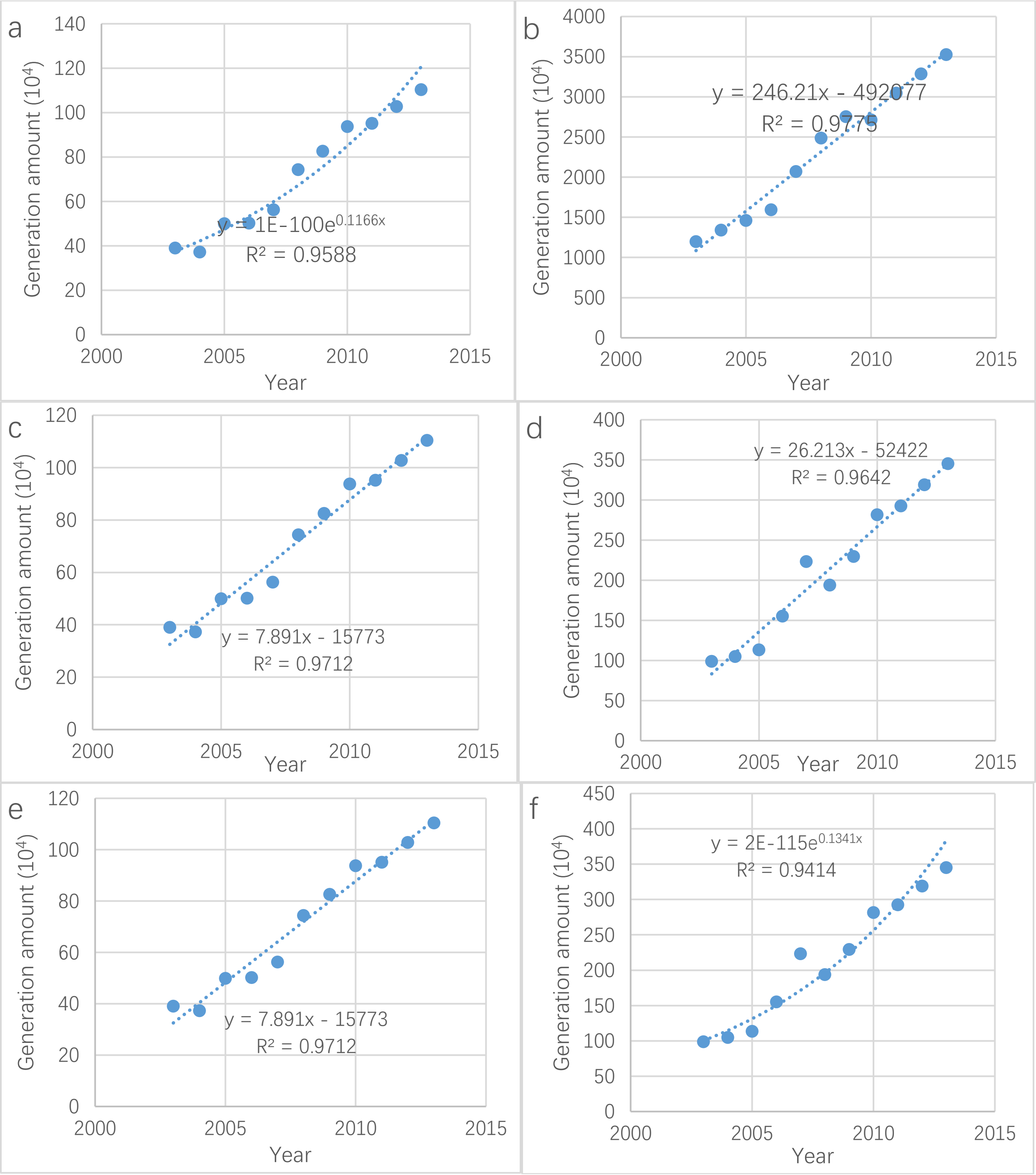

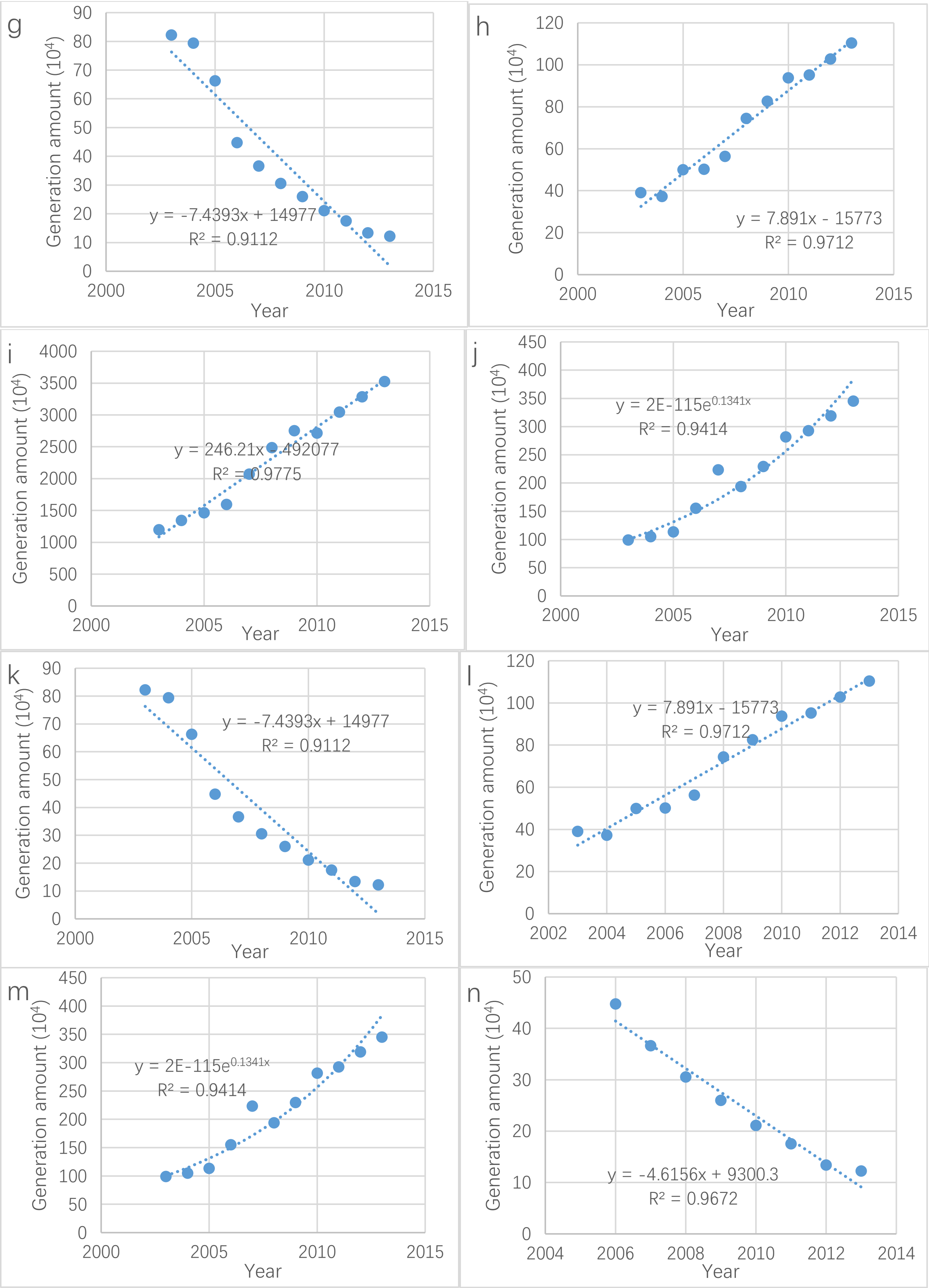

g
Generation amount (10^4)
y = -7.4393x + 14977
R² = 0.9112
Year
h
Generation amount (10^4)
y = 7.891x - 15773
R² = 0.9712
Year
i
Generation amount (10^4)
y = 246.21x - 492077
R² = 0.9775
Year
j
Generation amount (10^4)
y = 2E-115e^0.1341x
R² = 0.9414
Year
k
Generation amount (10^4)
y = -7.4393x + 14977
R² = 0.9112
Year
l
Generation amount (10^4)
y = 7.891x - 15773
R² = 0.9712
Year
m
Generation amount (10^4)
y = 2E-115e^0.1341x
R² = 0.9414
Year
n
Generation amount (10^4)
y = -4.6156x + 9300.3
R² = 0.9672
Year

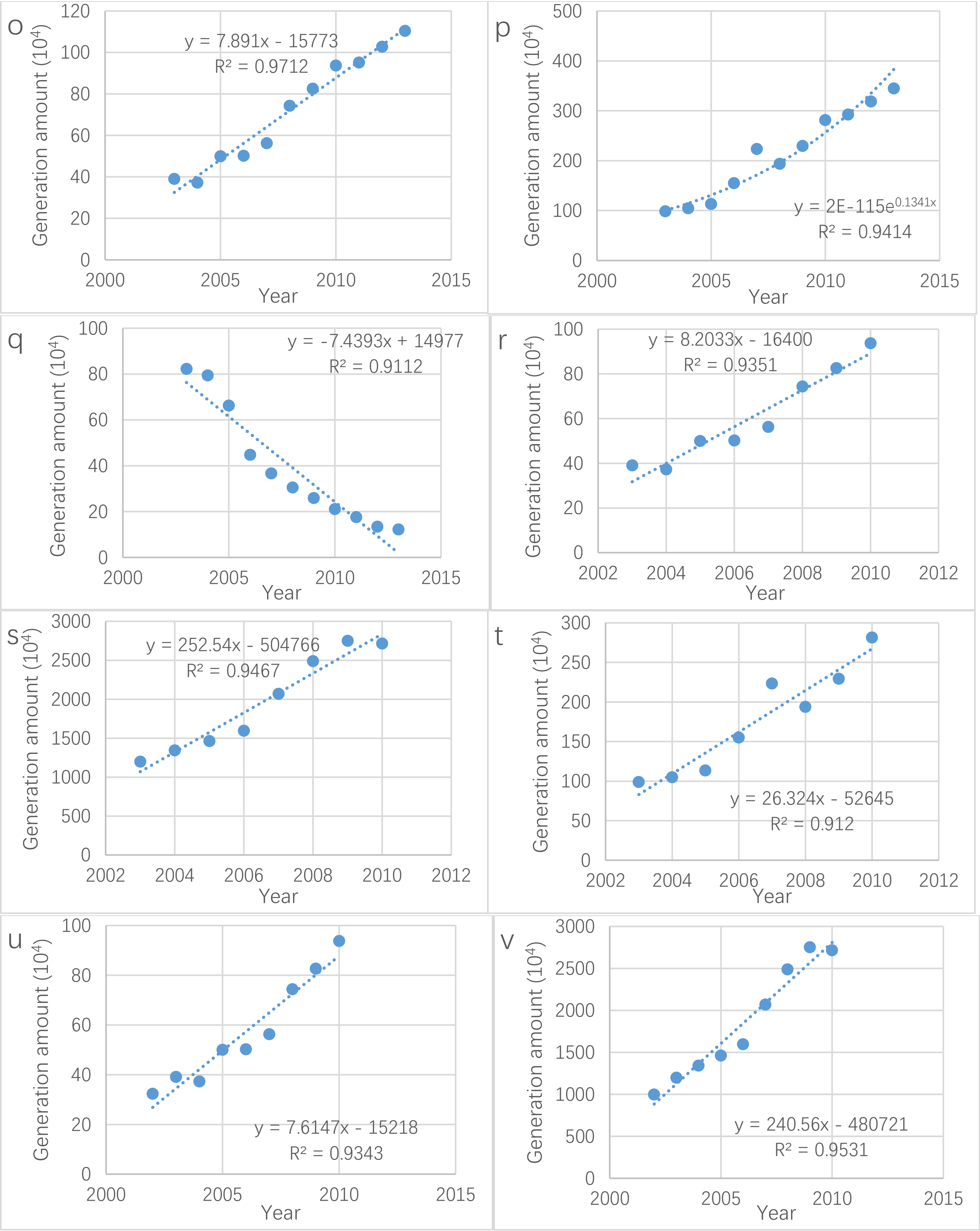
o
Generation amount (10^4)
Year
y = 7.891x - 15773
R² = 0.9712
p
Generation amount (10^4)
Year
y = 2E-115e^0.1341x
R² = 0.9414
q
Generation amount (10^4)
Year
y = -7.4393x + 14977
R² = 0.9112
r
Generation amount (10^4)
Year
y = 8.2033x - 16400
R² = 0.9351
s
Generation amount (10^4)
Year
y = 252.54x - 504766
R² = 0.9467
t
Generation amount (10^4)
Year
y = 26.324x - 52645
R² = 0.912
u
Generation amount (10^4)
Year
y = 7.6147x - 15218
R² = 0.9343
v
Generation amount (10^4)
Year
y = 240.56x - 480721
R² = 0.9531

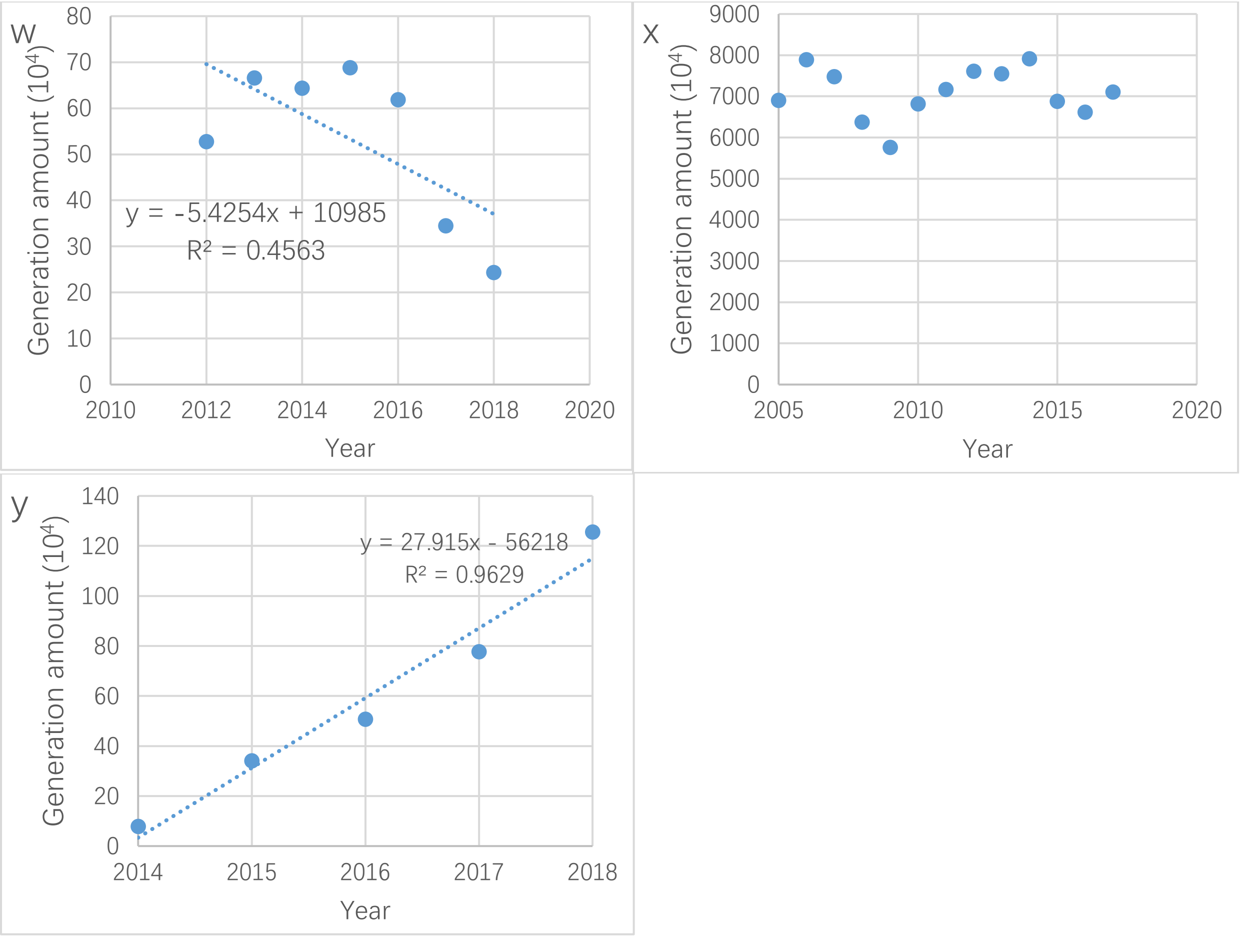

Note: Ideally, the "inverted-U" shape can describe the relationship between resource consumption and economic growth.[6] It is characterized of the rapid growth at the start stage, stable growth at the middle stage, and slow growth for a constant at the late stage, which is approximately expressed by exponential function, linear function, and constant value, respectively.[7] Accordingly, emerging product will likely fit for exponential function and linear function. Experienced product will possible keep constant like bicycle or decline like CRT-TV.

Supplementary Fig. 9 Linear regression of imported scraps for I'(x)

a Copper scrap; **b** Aluminum scrap; **c** Iron scrap; **d** Plastics scrap

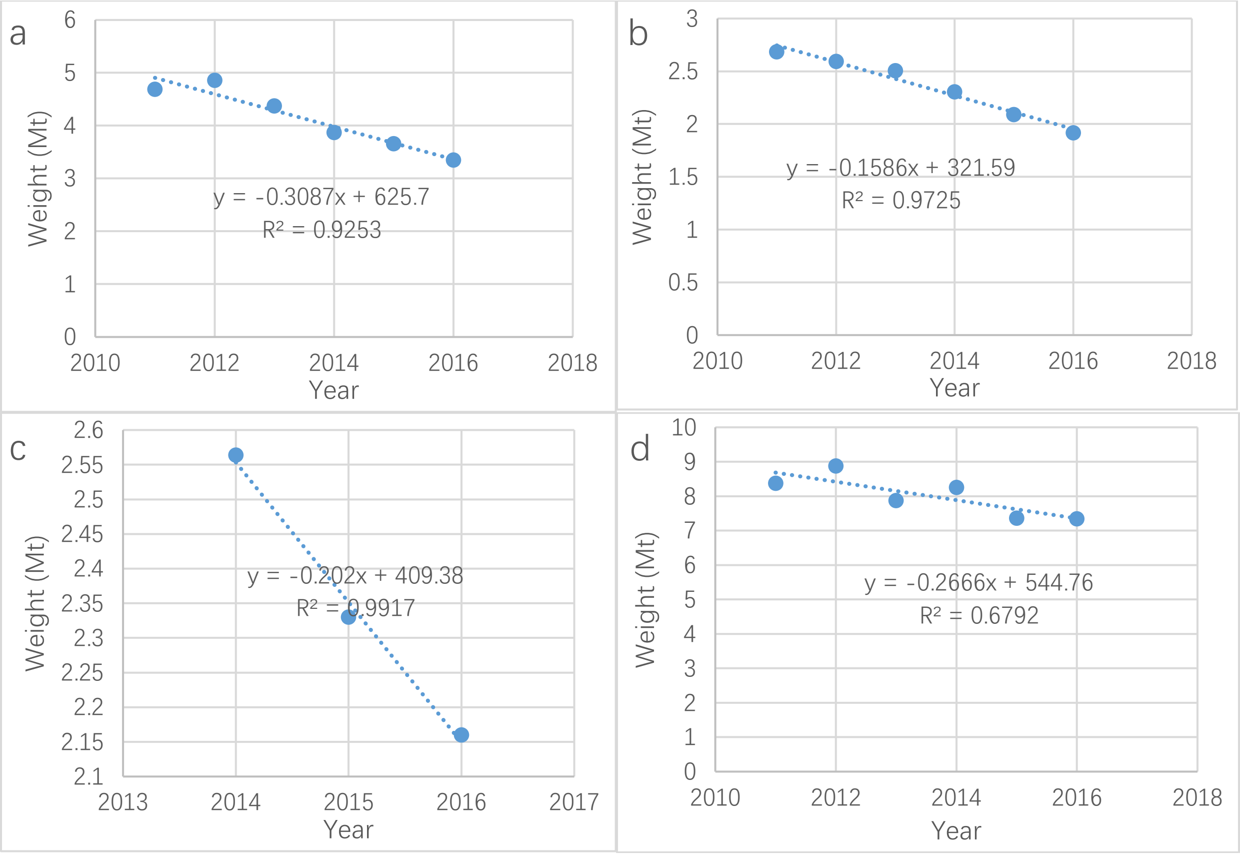

Supplementary Fig. 10 Estimation of China's AM from 2010 to 2050: Range for *D*(*x*) (Mt)

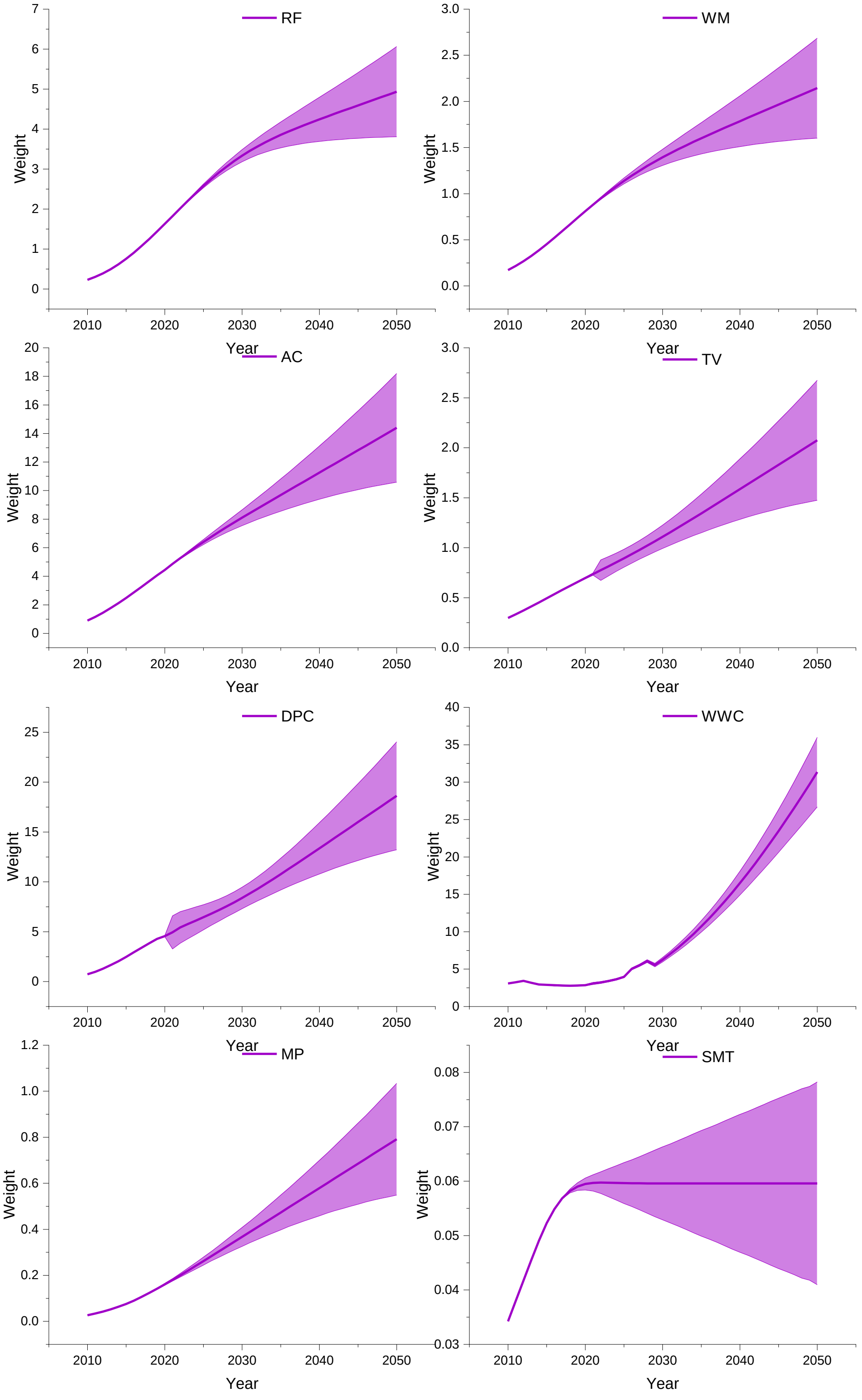

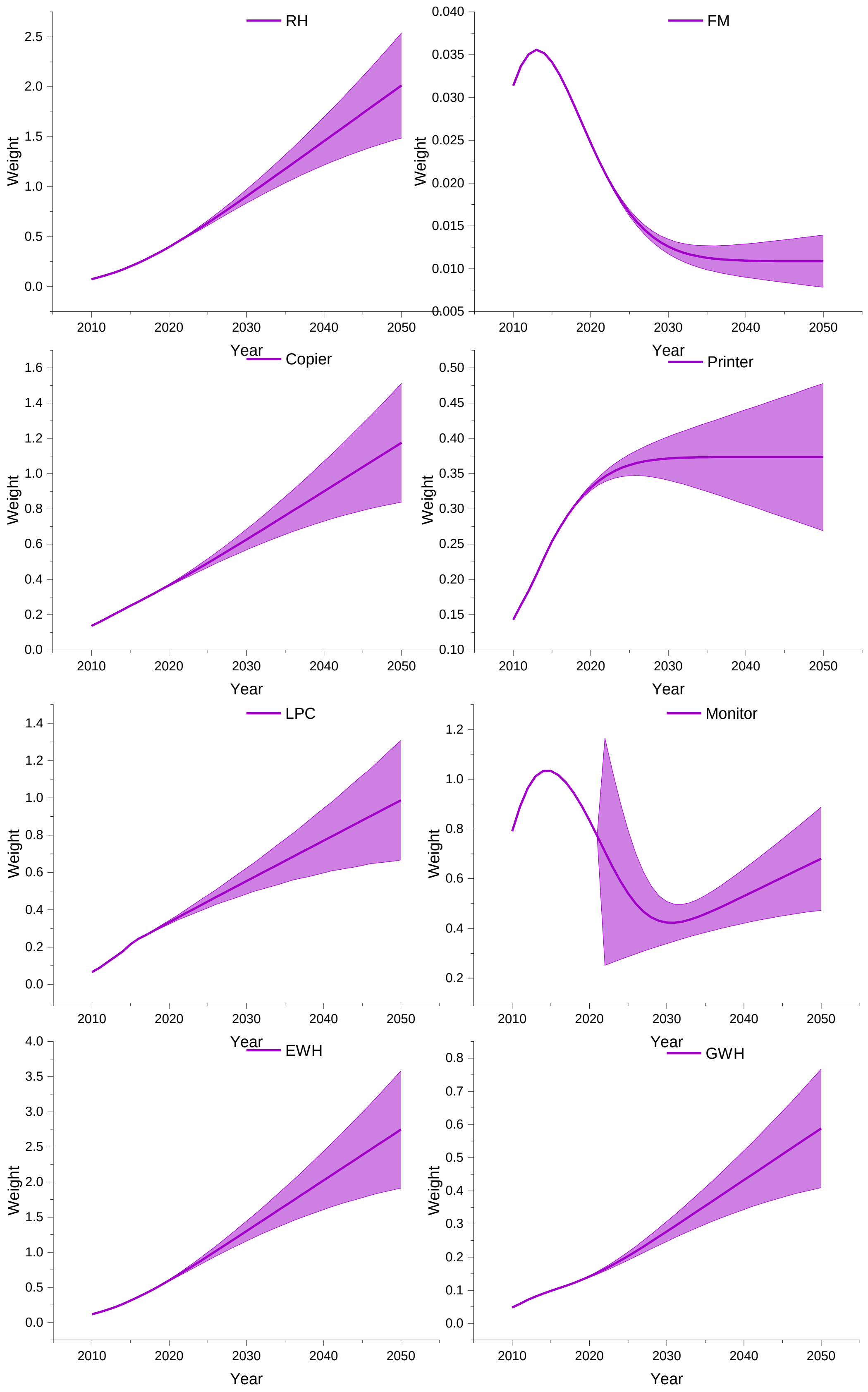
RH
FM
Copier
Printer
LPC
Monitor
EWH
GWH
Weight
Year
2010
2020
2030
2040
2050

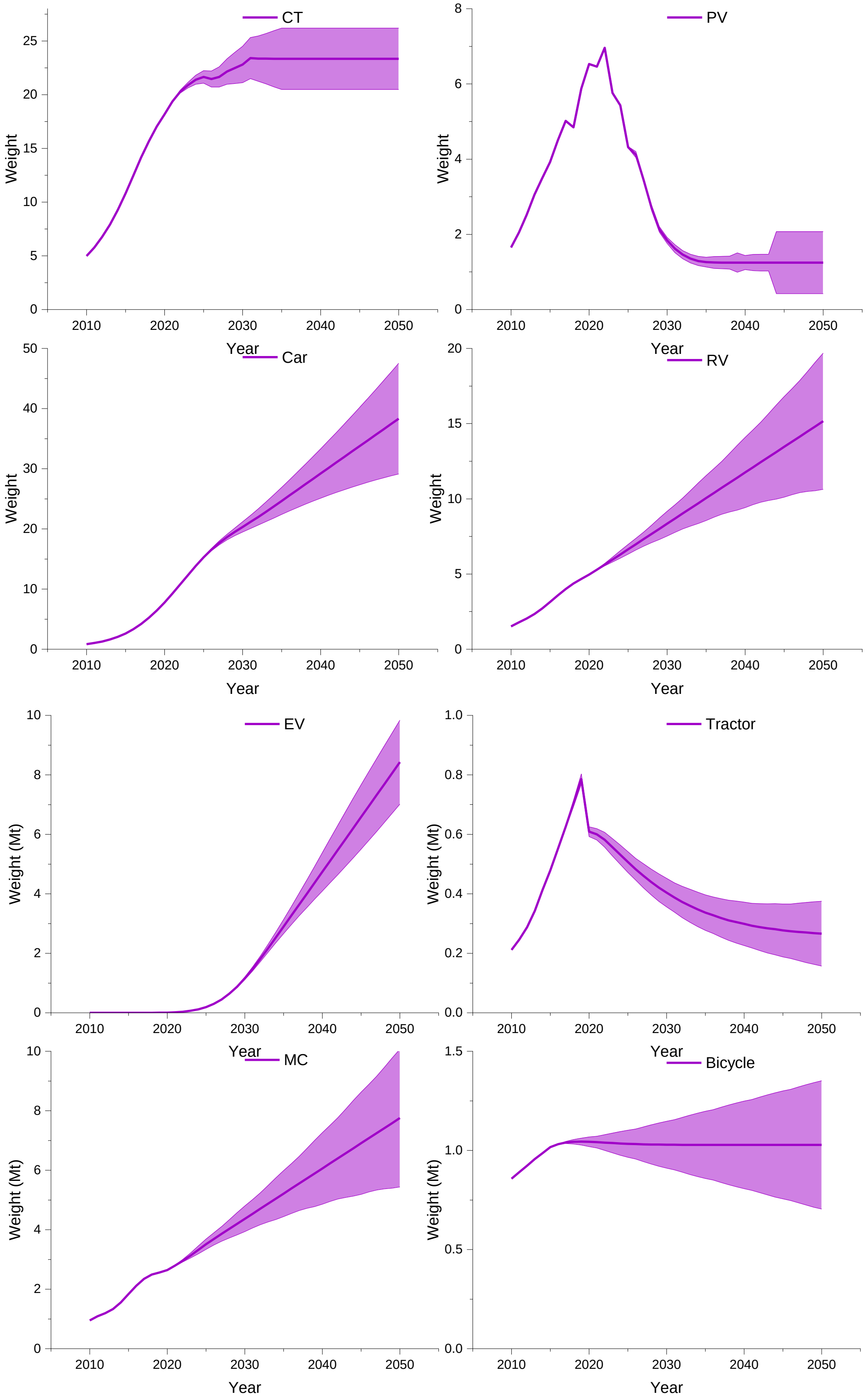
CT
Weight
Year
PV
Weight
Year
Car
Weight
Year
RV
Weight
Year
EV
Weight (Mt)
Year
Tractor
Weight (Mt)
Year
MC
Weight (Mt)
Year
Bicycle
Weight (Mt)
Year

Supplementary Fig. 11 Resources weight in yearly-generated three AMs for $D_{m,j}$

a base materials in WEEE; **b** precious metals, rare metals, and rare earth in WEEE; **c** base materials in ELV; **d** Zn, Co, W, Pb, and Mg in ELV; **e** precious metals in ELV; **f** base materials in WWC.

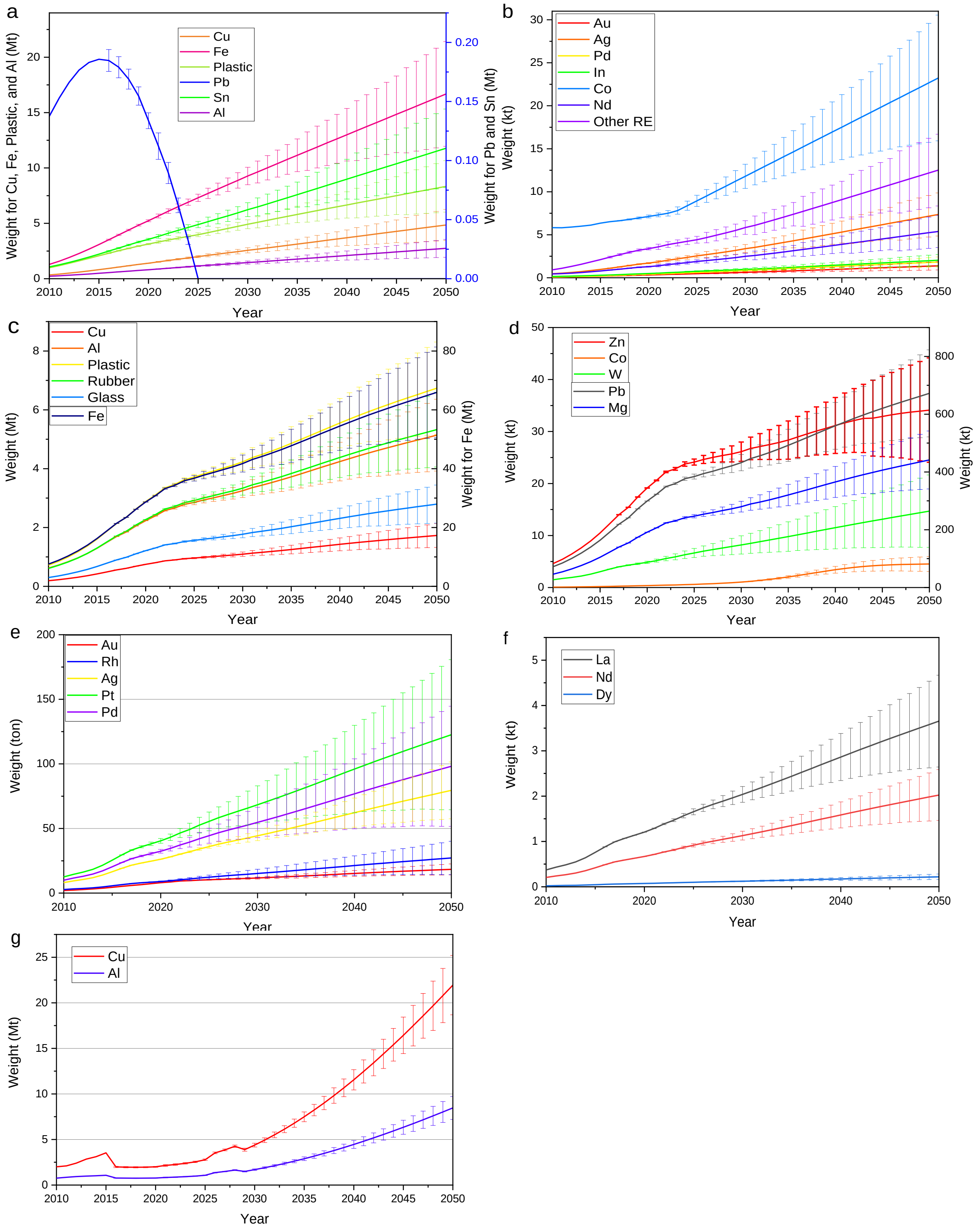

Supplementary Fig. 12 Relative economic shares of materials in total AM

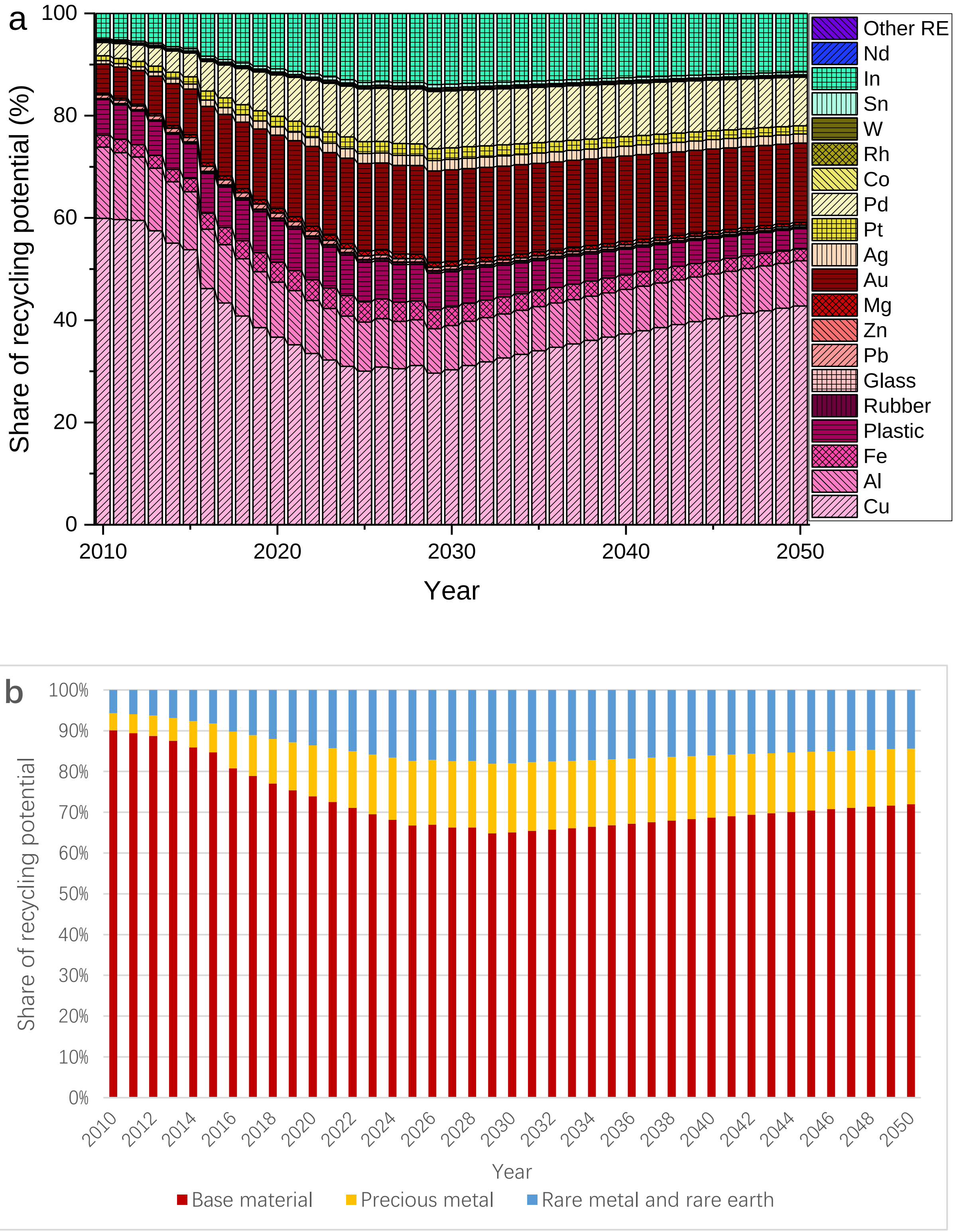

Note: Base material: Cu, Al, Fe, Plastic, Rubber, Glass, Pb, Zn, Mg, Co, and Sn; Precious metal: Au, Ag, Pt, Pd, and Rh; Rare metal and rare earth (RE): W, In, Nd, Dy, La, and other RE.

Supplementary Fig. 13 The comparison of typical AM estimation

a Typical WEEE quantity; **b** Typical WEEE weight; **c** Typical ELV quantity.

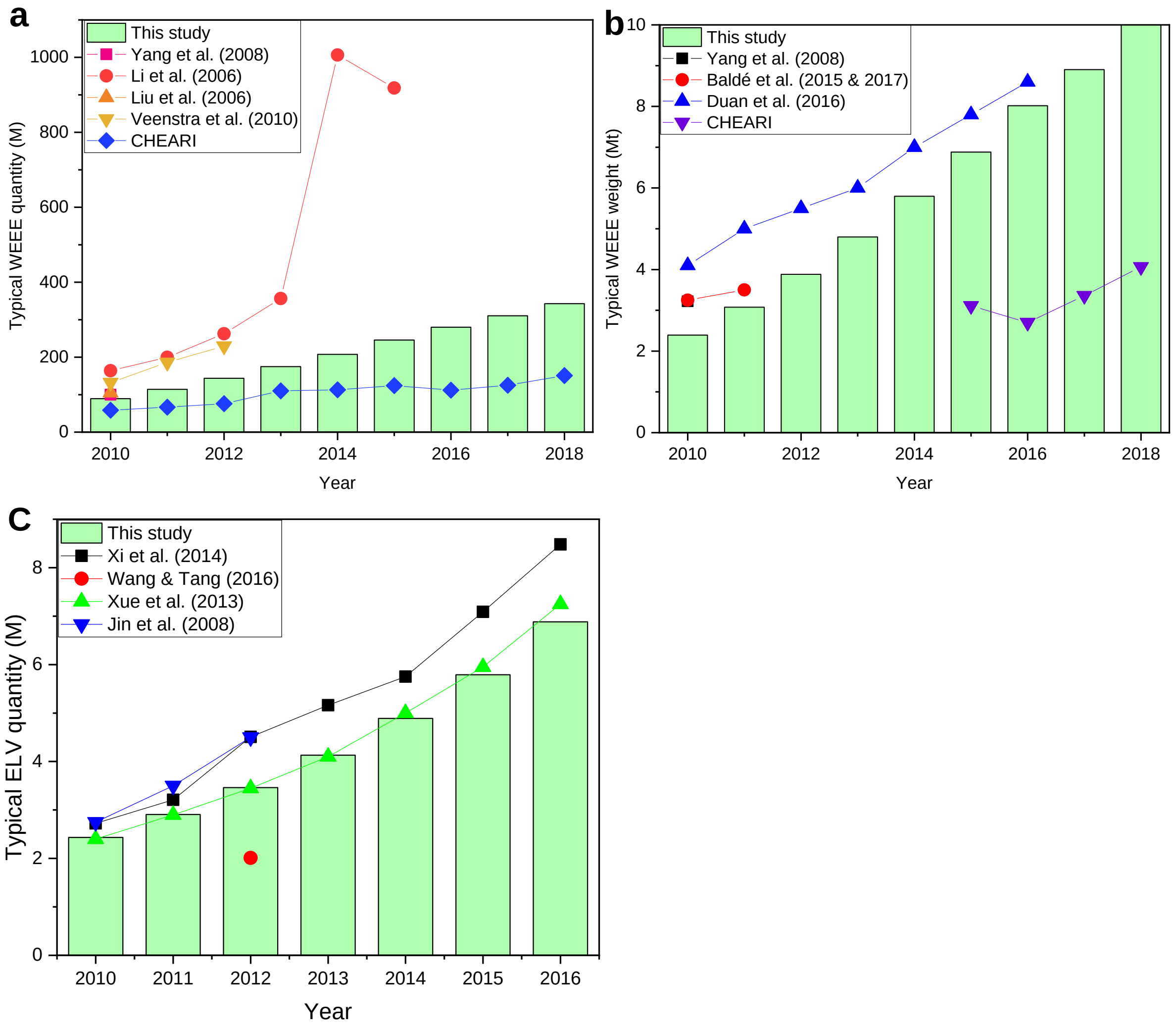

Note: Typical WEEE covered RF, WM, TV, PC, and AC; typical ELV covered PV, CT, car, and RV. Yang et al. (2008) [8], Li et al. (2006) [9], Liu et al. (2006) [10], Veenstra et al. (2010) [11], Baldé et al. (2015 & 2017) [12, 13], Duan et al. (2016) [14], Xi et al. (2014)[15], Xue et al (2013)[16], Jin et al. (2008)[17], and CHEARI[18, 19, 20, 21, 22].

Supplementary Fig. 14 Validation for all the registered vehicles quantity

a Comparison of estimated value in this study to real-world value; **b** significance test for real value and estimated value.

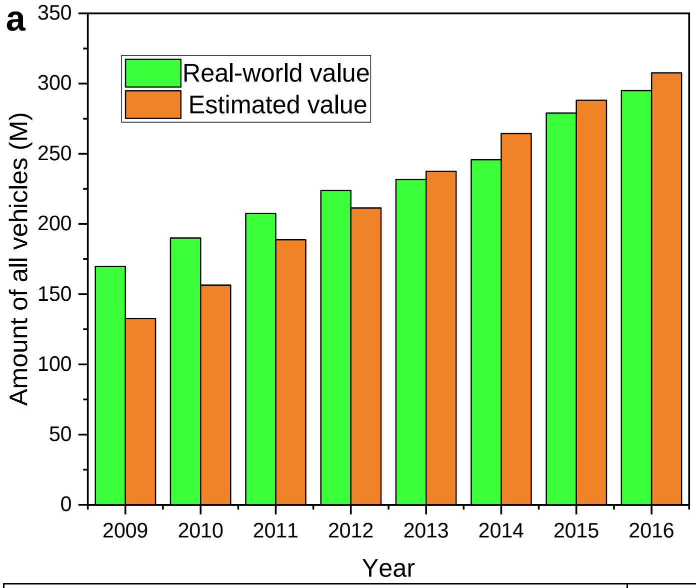

**b**	*t* Statistic	Degrees of freedom	Prob>|*t*|
Equal Variance Assumed	0.26019	14	0.79851
Equal Variance NOT Assumed (Welch Correction)	0.26019	12.3478	0.79901

Note: At 0.05 level, when equal variance is assumed, Mean 1 (real value) – Mean 2 (estimated value) is NOT significantly different from 0. And at 0.05 level, when variance is not assumed, Mean 1 (real value) – Mean 2 (estimated value) is NOT significantly different from 0.

Supplementary Table 1 *P*(*x*): Statistics of domestic production for EEE, vehicle, and wiring & cable in China

a EEE for the year of 1990-2016 (million unit, M)

Year	Refrigerator	Washing machine	Air conditioner	TV	Desktop PC	Laptop PC	Mobile phone	Camera	Digital camera	SMT	Fax machine	Copier	Printer	Monitor	Range hood	Electric water-heater	Gas water-heater
1990	4.6306	6.6268	0.2407	10.3304	0.0821	0	0	0	0	0	0	0	0	0	0	0	0
1991	4.6994	6.8717	0.6303	12.0506	0.1625	0	0	0	0	0	0	0	0	0	0	0	0
1992	4.8576	7.0793	1.5803	13.3308	0.1262	0	0	0	0	0	0	0	0	0	0	0	0
1993	5.9666	8.9585	3.4641	14.3576	0.1466	0	0	0	0	0	0	0	0	0	0	0	0
1994	7.6812	10.9424	3.9342	16.8915	0.2457	0	0	0	0	0	0	0	0	0	0	0	0
1995	9.1854	9.4841	6.8256	20.5774	0.8357	0	0	0	0	0	1.361	0	0	0	0	0	0
1996	9.7965	10.7472	7.8621	25.376	1.3883	0	0	0	0	0	1.379	0	0	0	0	0	0
1997	10.4443	12.5448	9.7401	27.1133	2.0655	0	0	0	0	0	1.625	0	0	0	0	0	0
1998	10.6	12.0731	11.5687	34.97	2.914	0	22.152	55.2187	0	0	1.287	0	0	0	0	0	0
1999	12.1	13.4217	13.3764	42.62	4.05	0	32.03	48.3229	0	0	1.6	0	0	0	0	0	0
2000	12.79	14.4298	18.2667	39.36	6.72	0	52.479	55.1452	0	39.36	1.9629	1.5663	17.991091	34.5092	3.6615	0	0
2001	13.5126	13.4161	23.3364	40.937	8.7765	0	80.317	59.6209	0	40.937	3.1819	1.4412	20.639945	38.2779	4.5628	1.533117	2.046028
2002	15.9887	15.9576	31.3511	51.55	14.6351	0	121.4635	53.0961	0	51.55	2.9729	2.0739	20.710473	52.2556	4.2646	2.091307	2.517558
2003	22.4256	19.6446	48.2086	65.414	32.167	14.352	182.3137	61.9814	0	65.414	7.4658	2.6417	30.68809	84.6903	4.7725	3.936664	3.273062
2004	30.0759	25.3341	63.9033	74.318	59.749	32.3838	237.5158	78.914	42.0759	74.318	8.5116	3.2457	49.769881	145.3358	6.7744	5.30086	5.302679
2005	29.8706	30.3552	67.6457	82.8322	80.8489	45.6499	303.5421	81.99	55.2297	82.8322	10.6815	4.0356	40.929867	105.1967	7.301	6.8243791	7.028524
2006	35.3089	35.605	68.4942	83.754	93.3644	62.4937	480.1379	85.5151	66.9511	83.754	11.8863	4.678	46.4027	133.5998	11.1807	10.765845	8.235657
2007	43.9713	40.051	80.1428	84.7801	120.7338	86.7143	548.5786	86.896	74.9346	84.7801	8.8853	4.5236	42.3469	144.3812	12.1688	15.175366	9.712175
2008	47.9995	44.47	81.474	91.871	158.5365	108.5868	559.451	89	81.8826	91.871	7.6991	5.8419	43.3398	133.6457	16.3315	17.196775	10.87103
2009	59.3045	49.7363	80.7825	98.9879	182.1507	150.0947	619.2447	84.5781	80.2632	98.9879	6.8351	4.2102	36.4082	131.2347	17.1448	14.682645	14.246062
2010	72.9572	62.4773	108.8747	118.3003	245.8446	185.8412	998.2736	93.277	91.285	118.3003	1.8109	5.3482	60.6876	139.2699	20.2833	18.728117	13.332711
2011	86.992	67.1594	139.125	122.3134	320.3693	238.9741	1132.5771	82.4134	80.5125	122.3134	2.6813	6.5505	55.1833	126.8054	20.3206	24.178	15.688
2012	84.27	67.9112	132.811	128.235	354.110	252.893	1181.545	88.0171	70.0707	128.235	2.6357	6.096	70.59	127.132	22.353	25.1263	11.2126

				2	2	7	7			2		9	21	7	7		
2013	92.6102	72.019	130.572	127.7605	336.61	272.7885	1455.61	46.9089	36.2014	127.7605	1.7208	6.354	73.7914	132.57	25.594	34.9255	12.023
2014	87.961		144.633	141.289	312.32	367.89	1712.6	31.23	24.675	122.8678	1.488	7.1286	66.011		30.8225	34.2968	14.7666
2015	79.9275	72.745	153.582	144.7573	389.02	312.59	1969.59	28.454	19.227	116.647	1.652	7.342	55.44		30.4639	36.355	15
2016	92.383	76.209	160.493	174.834	331.443	290.085	2261.087	23.0677	15.2727		1.763						17.7885

Source: http://data.stats.gov.cn/workspace/index?m=hgnd; http://mcin.macrochina.com.cn/index.shtml?ny=1.

b Vehicle for the year of 1991-2015 (M)

Year	Car			PV				CT				RV	MC	Tractor*	Bicycle	EV
	Capacity≤ 1L	Capacity: 1-1.6L	Capacity>1.6L	Heavy	Middle	Small	Mini	Heavy	Middle	Small	Mini					
1991				0.00301	0.021417	0.131501	0.019814	0.019026	0.203951	0.179327	0.049719					0
1992				0.004407	0.033013	0.193899	0.041263	0.025391	0.259795	0.278526	0.062702					0
1993				0.00473	0.024367	0.191194	0.071922	0.033782	0.334656	0.330685	0.075745					0
1994				0.004062	0.019503	0.189272	0.104322	0.037173	0.313308	0.334706	0.100689					0
1995				0.003863	0.02274	0.22531	0.153541	0.030854	0.247982	0.331517	0.111469			0.063		0
1996				0.003632	0.018624	0.197549	0.175387	0.031655	0.211789	0.303422	0.141748			0.084		0
1997				0.004663	0.01593	0.189723	0.225299	0.030904	0.185726	0.297091	0.145597			0.082		0
1998				0.004215	0.011858	0.125502	0.179525	0.034829	0.183617	0.297351	0.145904			0.0678	23.1249	0
1999				0.006438	0.024562	0.154223	0.239677	0.047127	0.184738	0.386841	0.137606			0.0654	23.9757	0
2000				0.0064	0.029509	0.207579	0.334411	0.082146	0.146028	0.388831	0.134694			0.041	29.0679	0
2001				0.010028	0.046143	0.24032	0.424697	0.157405	0.151929	0.363234	0.130508	0.323372	9.959837	0.0382	29.0226	0
2002				0.014536	0.054294	0.279391	0.549422	0.255406	0.163902	0.525208	0.14803	0.390214	11.9857	0.0454	39.5752	0
2003	0.075697	1.005963	0.98914	0.019136	0.052128	0.435149	0.644553	0.261688	0.136218	0.687877	0.142374	0.372528	13.42259	0.0488	54.517	0
2004	0.159447	1.067311	1.049542	0.025435	0.0515	0.394235	0.744379	0.379713	0.161888	0.806607	0.166661	0.499501	14.61822	0.1138	79.0622	0
2005	0.197953	1.437731	1.134416	0.025617	0.056377	0.4775	0.736226	0.225951	0.198657	0.850102	0.235183	0.501938	15.95509	0.1633	69.0064	0
2006	0.199744	2.117571	1.552085	0.02908	0.059822	0.595112	0.844746	0.303592	0.201821	0.949368	0.298192	0.562731	20.69447	0.1993	78.8663	0
2007	0.225143	2.338379	2.234278	0.037447	0.071215	0.734243	0.906284	0.489883	0.23455	1.123163	0.309739	0.7435335	24.87791	0.2031	74.752	0
2008	0.27216	2.826435	1.939505	0.025993	0.047493	0.525344	0.650244	0.53604	0.206349	1.169278	0.35854	0.825816	27.51964	0.2844	63.7487	0
2009	0.516735	4.673203	2.294862	0.030239	0.051774	0.721923	1.272018	0.639885	0.269601	1.622068	0.517616	0.9375682	27.14695	0.3713	57.5765	0
2010	0.761608	5.998239	2.816053	0.036573	0.048826	0.150934	2.28352	1.058635	0.268982	1.972937	0.619809	1.119563	27.12393	0.3368	68.1948	0
2011				0.034522	0.042773	0.149679	2.044927	0.822696	0.294448	1.843632	0.490196		27.355	0.4019	71.6911	0.008368
2012				0.040923	0.046391	0.174186	2.456	0.59106	0.288719	1.827667	0.534284		26.0302	0.5273	76.1285	0.012552
2013				0.02026	0.022604	0.096539	1.481497	0.760581	0.285461	1.894993	0.527466		25.271	0.6656	75.4525	0.017533
2014				0.016468	0.015543	0.087754	1.467236	0.747451	0.247899	1.661643	0.538908		26.917	0.6437	79.1014	0.078499
2015				0.006819	0.00524	0.029915	0.762226	0.37055	0.114381	1.467718	0.538688		25.028	0.6882	68.8224	0.340471

Note: * big and medium size tractor.

c Wiring & cable for the year of 1996-2014 (M)

Year	Power cable (ton)	Aluminum cable copper reinforced (ton)	Optical communication cable (ton)	Telecommunication cable (ton)	Electromagnetic wire (ton)	Power cable (km)
1996	0.4					
1997	0.5					
1998	0.6					
1999	0.7					
2000	0.9					
2001	1.1074	0.5525				
2002	1.27355	0.663				
2003	1.51225	0.5729				
2004	2.34345	0.595				
2005	4.0621	0.7934				
2006	6.13955	0.926	3.799035	0.546176		
2007	6.8651	1.0776	5.738175	0.7015808		
2008	9.8214835	0.7092479	7.279485	0.6829728		
2009	10.693349	0.7379015	12.64163	0.4667144	1.15	
2010	13.6568295		14.13519	0.4153856	1.26	
2011	15.39685		20.69558	0.4354152	1.2843	27.0548
2012	20.00985		27.76274	0.3694528	1.3543	30.7937
2013	20.97332		34.08989	0.3460592	1.45	40.0197
2014	27.8518435		45.55677	0.3941632	1.55	55.7037

Supplementary Table 2 *I*(*x*) and *E*(*x*): Statistics of importation and exportation in China

a Product (M)

Year	EEE								Vehicle					Wiring & cable
	TV		SMT		MP		Camera	Copier	Truck	Automobile		Car	Bicycle	
	Importation	Exportation	Importation	Exportation	Importation	Exportation	Exportation	Importation	Importation	Importation	Exportation	Importation	Exportation	Exportation (ton)
1990	0.67						4.19							
1991	0.33						8.26		0.018578	0.098454		0.054409	7.29	
1992	0.23						29.23		0.041521	0.209992		0.027558	10.24	
1993	0.77						38.82		0.07257	0.310096		0.071125	10.32	
1994	0.75	10.79		72.6			43.21		0.067631	0.281425		0.034643	13.42	0.163
1995	1.16	11.51		76.12			61.5		0.011728	0.158115		0.023802	12.62	0.1985
1996	0.42	10.32		85.71			61.8		0.005916	0.07536	0.015	0.057942	12.17	0.2296
1997	0.43	9.06		100.11			65.59		0.006855	0.048441	0.0148	0.031944	14.39	0.3034
1998	0.31	10.21		106.86			67.33		0.004372	0.039711	0.0115	0.018046	17.61	0.3634
1999	0.26	12.98		134.22			59.51		0.002682	0.034906	0.008	0.019952	22.7	0.4382
2000	0.1	19.44		160.3	0.023712	160.3	90.73		0.003085	0.042371	0.0228	0.021614	32.86	0.5341
2001	0.09	21.03		148.97	0.037397	148.97	86.93		0.003138	0.072047	0.0248	0.046632	34.94	0.5447
2002	0.17	31.64		168.81	0.037674	168.8	91.56		0.006692	0.127367	0.0435	0.070326	45.56	0.6901
2003	1.01	47.62		193.27	0.033154	193.27	61.21		0.009769	0.172339	0.1322	0.103017	50.44	0.848
2004	0.78	63.09		199.02	0.027458	199	56.31		0.007989	0.175914	0.4014	0.116085	51.75	1.0771
2005	0.71	85.92		198.06	0.035923	198.06	45.93	1.025185	0.002928	0.16302	1.0809	0.076542	53.85	1.2341
2006	1.42	95.29	62.748928	693.2	0.036281	693.2	35.1	0.905703	0.00545	0.226922	0.3462	0.11178	55.99	1.4092
2007	1.21	51.03	18.734975	677.49	18.734975	677.49	21.25		0.007659	0.312085	0.61	0.139867	59.23	1.6172
2008	0.64	51.38	20.071491	700.23	20.071491	700.23	123.64		0.008719	0.40753	0.64	0.154521	56.59	1.69
2009	0.15	55.64	26.431385	712.11	26.431385	712.11	117.78		0.007143	0.418867	0.35	0.164837	46.11	1.43
2010	0.03	67.23	20.698023	903.95	20.698023	903.95	128.5		0.01335	0.810085	0.54	0.343653	58.16	1.74
2011	0.02	65.7	11.443807	1006.27	11.443807	1006.27	113.98		0.018034	1.035557	0.82	0.41027	55.72	1.8
2012	0.03	61.57	11.6741	1129.51	11.6741	1129.51	100.93		0.017998	1.129731	0.99	0.446783	57.15	1.86
2013	0.05	59.62	10.016117	1295.22	10.016117	1295.22	68.41		0.009418	1.192179	0.92	0.423399	56.95	1.95
2014	0.0606	74.0336	11.880918	1421.2255		1334	56.7588		0.009589	1.422717	0.8971	0.469617	62.655	2.1732
2015	0.0585	71.8342	18.492645	1445.5739			51.5947		0.004883	1.097418	0.7228	0.352461	57.8133	2.1675
2016							38.05							

Source: http://cndata.datesdata.com.cn/index.aspx;
http://data.stats.gov.cn/workspace/index;jsessionid=8506E2D79578DAE2FC42D18AE7790A9A?m=hgnd .

b Imported AM

Year	WEEE (kt) [5]		Copper scrap (Mt)	Aluminum scrap (Mt)	Steel scrap (Mt)	Plastics scrap (Mt)
	Estimation	Range*	Value	Value	Value	Value
2005			4.82	1.69		
2006			4.94	1.77		
2007			5.58	2.09	3.394	7
2008			5.58	2.15	5.589	7.07
2009			4	2.63	13.69	7.33
2010	877	[600-1500]	4.36	2.85	5.848	8.01
2011	808	[600-1500]	4.69	2.69	6.766	8.38
2012	738	[600-1500]	4.86	2.59	4.974	8.88
2013	669	[600-1500]	4.37	2.50	4.465	7.88
2014	600	600	3.87	2.31	2.564	8.26
2015	531	[0-600]	3.66	2.09	2.33	7.36
2016	462	[0-600]	3.35	1.92	2.16	7.35

Note: data of copper, aluminum, steel, and plastics scrap from National Statistics (http://data.stats.gov.cn/english/)

Supplementary Table 3 w_i: Weights of each product and those data distributions used for Monte Carlo simulation

a EEE (kg) [5]

Type	Mean	Stand. Dev.	Prob. Dist.	Type	Mean	Stand. Dev.	Prob. Dist.
CRT-TV	27.81	12.01	Beta	MP	0.100	0.023	Beta
CRT-BTV	10.274	1.752	Beta	SMT	0.498	0.114	Beta
RF	40.09	8.79	Beta	RH	23.57	4.21	Beta
MF	9.006	2.681	Beta	FM	6.785	2.437	Beta
LPC	1.858	0.681	Beta	Copier	60.80	37.77	Beta
WM	18.00	3.53	Beta	Printer	6.284	3.448	Beta
CRT-M	13.45	2.58	Beta	EWH	24.36	4.48	Beta
LCD-M	4.885	2.113	Beta	GWH	12.42	2.68	Beta
AC	44.90	20.40	Beta				

Note: LCD-TV: TV with liquid crystal display (LCD); CRT-CTV: color TV with cathode-ray tube (CRT); CRT-BTV: black TV with CRT; RF: refrigerator; MF: mainframe; LPC: laptop personal computer; WM: washing machine; CRT-M: CRT monitor used for mainframe; LCD-M: LCD monitor used for mainframe; AC: air conditioner; DPC: desktop personal computer; MP: mobile phone; SMT: single-machine telephone; RH: range hood; FM: fax machine; EWH: electric water-heater; GWH: gas water-heater.

b Vehicle[23, 24]**(ton)**

Type	Mean	Range	Prob. Dist.	Type	Mean	Range	Prob. Dist.
LPV	14	[11-16]	Beta	Scar	0.5	[0-1]	Beta
MPV	8	[4-11]	Beta	Mcar	1.2	[1-1.4]	Beta
SPV	3	[2-4]	Beta	Lcar	2.2	[1.4-3]	Beta
MiPV	1	[0-2]	Beta	RV	4	[3-5]	Beta
HCT	16	[14-18]	Beta	EV	1.5	[1-2]	Beta
MCT	10	[6-14]	Beta	MC	0.11	[0.1-0.12]	Beta
SCT	3.4	[1.8-6]	Beta	Tractor	1.6	[1.2-1.9]	Beta
MiCT	0.9	[0-1.8]	Beta	Bicycle	0.015	[0.012-0.018]	Beta

Note: LPV: large passenger vehicle; MPV: medium passenger vehicle; SPV: small passenger vehicle; MiPV: mini passenger vehicle; HCT: heavy cargo truck; MCT: medium cargo truck; SCT: small cargo truck; MiCT: mini cargo truck; Scar: car with capacity⩽1L; Mcar: car with capacity of 1-1.6L; Lcar: car with capacity>1.6L; RV: refit vehicle; MC: motorcycle; EV: electric vehicle.

Supplementary Table 4 c_{ij}: Average content of resources contained in various products

a WEEE [25, 26, 27, 28, 29, 30, 31, 32, 33, 34, 35, 36, 37, 38, 39, 40]

Type	Valuable metals (%)			Precious metals (10^{-6})			Rare metals (10^{-6})		Rare earths (10^{-6})			Plastic	Glass
	Cu	Al	Fe	Au	Ag	Pd	In	Co	Nd	Y	Eu	(%)	(%)[#]
RF	3.4	1.1	50	0	0	0	0	0	0	0	0	43.3	2
WM	4	3	53	0	0	0	0	0	0	0	0	26	1
AC	18.5	7	45.9	0	0	0	0	0	0	0	0	17.5	0
CRT-TV	3	2	10	1.4	19.6	0.7	0	0	0	67.51[*]	5.47[*]	23	45
LCD-TV	1	4	30	0	0	0	102	0	0	0	0	40	18[41]
CRT-Desktop PC	6.5	2	26	46	207	18.4	0	0	170	4.40E-05[*]	3.56E-6[*]	23	40
LCD-Desktop PC	7.2	3.6	18	60	300	25	40	0	270	0	0	4.3	12
LPC	5.7	1.5	20	32	190	19	140	10700	360	0	0	16	20
MP	10.7	2.6	15	25	883	2.6	1102	3738	4500	39	42	25.6	10.6[42]
SMT	2	2	1	2.2	30.8	1.1	10000	0	0	0	0	69	0
FM	4	15	30	0	0	0	0	0	0	0	0	30	0
Copier	3.5	20	16	0	0	0	0	0	0	0	0	35.53	3
Printer	0.5	18	32	0	0	0	0	0	0	0	0	40	0
RH	10	8	30	0	0	0	0	0	0	0	0	5	2
EWH	5	10	35	0	0	0	0	0	0	0	0	5	2
GWH	8	10	30	0	0	0	0	0	0	0	0	5	2

Note: All the data is the average of all collected references; [*]Data source from Supplementary Table 4c. All the data is assumed to fit for normal distribution.
[#]Glass: 45% for CRT-TV was determined by China's recycler of e-waste recycling, and other data from personal estimation.

b WEEE: Tin content in the typical WEEE

Year	Country	WEEE	RF	WM	AC	TV	MP	Desktop PC	Laptop PC	Hardcopy peripherals	Tin solder weight (ton)	Tin weight in one-ton e-waste (kg)
		Each weight (kg)	40	18	44.9	27	0.1	20.45	1.875	40		
2015	China	Unit (M)	79.93	72.75	153.58	72.98	1969.59	467.14	312.59	62.78	84,952*	3.24
		Weight (Mt)	3.197	1.310	6.896	1.970	0.197	9.553	0.586	2.511		
2016	The U.S.	Unit (M)	10.9	16.41	6.87	39.68	226.17	17.87	45.22	26	3,812	1.05
		Weight (Mt)	0.436	0.295	0.308	1.071	0.023	0.365	0.085	1.040		
Average												2.14

Note: The data of each EEE weigh from Zeng et al. (2016)[43]; China's data for EEEs production amount from China Statistics; China's data for EEEs ship amount from China Statistics from www.statista.com; *361.9kt×48.6%×48.3%=84,952 ton[44]; 3,812 from USGS.

c WEEE: Content of other rare earths (Y and Eu) in CRT monitor (w.t. %)

Rare earth	Fluorescent powder							CRT**		
	Collected data						Average	CTV	BTV	PC
Y	12.35*	12.56*	13.53*	13.74[45]	19.83[46]	19.11[47]	15.19	0.006751111	8.10133E-06	4.40E-09
Eu	1.145*	1.13*	1.06*	1.374[46]	1.42[47]	-	1.23	0.000546667	0.000000656	3.56E-10

Note: *Data determined by Dr Quanyin Tan at Tsinghua University. **Data determined with the following information: each CRT contains 10-12g fluorescent powder [47] in around 27kg, 10kg, and 13.5kg for CRT-CTV, CRT-BTV, and CRT-M, respectively.

d Vehicle: collected data (w.t. %)[23, 48, 49, 50, 51, 52, 53, 54, 55, 56, 57, 58]

Resource	Collected data											Mean	Minimum	Maximum
Fe	71	69	64.3	65.4	68	60.6	70.4	68.3	68.76	65.4	67.7	60.6	67.2	71
Al	4	5	8	7.3	8	5.7	7.0	8.9				8	6.3	4

Cu	1.2	1										1	1.1	1.2
Pb	0.8											0.8	0.8	0.8
Zn	1	1										1	1	1
Plastics	6	7	9.3	9	12.1	10.8	9.1	8.17	9.3	7.8		6	8.86	12.1
Tire & rubber	4	4	5.6	5	5.1	3.42	5.6	4.2				3.42	4.62	5.6
Glass	3	3	2.7	3	2.9	3.1	2.9	2.8				2.7	2.93	3.1

Note: All the data is assumed to fit for normal distribution. The average weight of power battery was 275kg, 235kg, 550kg, and 1900kg for each plug-in passenger vehicle, plug-in commercial vehicle, pure electric passenger vehicle, and pure electric commercial vehicle, respectively (http://auto.gasgoo.com/News/2017/07/04062820282070016964C501.shtml).

e Vehicle: composition in various vehicle (%)

Resource	Cu	Al	Fe	Au	Pb	Plastic	Zn	Mg	Rubber	Glass	Co*
PV	1.8	5	69	0.00002	0.8	7	0.05	0.5	5.1	3	-
CT	1.8	5	68	0.00002	0.8	7	0.05	0.5	5.1	3	-
Car	1.8	5	69	0.00002	0.8	7	0.05	0.5	5.1	3	-
RV	1.8	5	68	0.00002	0.8	7	0.05	0.5	5.1	3	-
EV	2	5	68	0.00002	0.8	7	0.05	0.5	5.1	3	0.102
Tractor	1.2	5	68	0	0	7	0.05	0.5	6.6	1	-
MC	2	8	60	0.00002	0	7	0	0	9	3	-
Bicycle	1	10	82	0	0	1	0	0	6	0	-

Note: Data from Supplementary Table 4d and personal estimation. *Since cobalt is used in power battery for EV, its composition in EV will be 0.92%×60%×(275+235+550+1900)/(4×4000)= 0.102% [0.92% is the composition of cobalt in MnNiCo cathode material (http://wemedia.ifeng.com/10681129/wemedia.shtml); 60% is the cathode material composition in EV battery)[29]; (275+235+550+1900)/(4×4000) is the average battery weight share in each EV]; Cobalt content in other vehicle is given in Table 4g.

f Vehicle: Mass content of W, Pt, Pd, and Rh in each vehicle (g/car)

Material	Year	Region	Total consumption quantity (ton)	Vehicle share (%)	Vehicle production quantity (k)*	Consumption quantity in each vehicle (g)
W	2000	The U.S.	14,890[59]	4.8[59]	12,774	56
	2016	World	86,400[60]	23[60]	94,031	211.3
	Average					134
Pt	2013	World	97.2 ton		86,953	1.118
Pd			77.8 ton			0.895
Rh			21.6 ton			0.248

Note: *data source from https://www.bts.gov/archive/publications/national_transportation_statistics/table_01_23; In 2013, around 97.2 ton, 77.8 ton, and 21.6 ton were globally utilized for Pt, Pd, and Rh, respectively in vehicle industry (http://www.hysec.com/f/tsnr/[D2014]/2014-12/TSNR100/23/RR_3003029292.pdf)[61], which is used as catalyst to emission controlling. Because the world production quantity was 86,953,000 unit, each vehicle will consume 1.118g Pt, 0.895g Pd, and 0.248g Rh.

g Vehicle: Mass content of other critical metals in an average ELV (g/car)

**Metal**	min	average	max	**Metal**	min	average	max	**Metal**	min	average	max
**Dy**	1.443256	2.013372	3.23657	**Nd**	14.06395	18.4593	24.49419	**Ag**	0.477907	0.725	1.136047
**La**	24.44767	33.33721	45.83721	**Co**	23.67442	31.93023	44.88372	**Sn (%)**	0.0015	0.0019	0.0023

Note: personal calculation based on the data[62].

h Copper and aluminum consumption in wiring & cable (kt) [63, 64]

Year	1991	1992	1993	1994	1995	1996	1997	1998	1999	2000	2001	2002	2003	2004	2005	2006
Cu	700	780	840	950	1140	1230	1300	1430	1500	1720	2000	2100	2400	2850	3120	3540
Al	220	220	240	250	270	280	320	350	430	580	760	850	930	980	1020	1070

Supplementary Table 5 Resource market prices in recent years (US$/ton)

Resource	Min	Max	Mean	Error
Ag	0.5×10^6	1.55×10^6	1.03×10^6	$\pm0.53\times10^6$
Al	1.3×10^3	3.3×10^3	2.3×10^3	$\pm1\times10^3$
Au	34×10^6	60×10^6	47×10^6	$\pm13\times10^6$
Co	2.3×10^4	5.2×10^4	3.75×10^4	$\pm1.45\times10^4$
Cu	2.8×10^3	1×10^4	6.4×10^3	$\pm3.6\times10^3$
Dy	2.62×10^5	2.92×10^5	2.77×10^5	$\pm3\times10^4$
Fe	51	188	120	±68
Glass	26	46	36	±10
In	13×10^6	35×10^6	24×10^6	$\pm11\times10^6$
La	4.6×10^3	5.4×10^3	6.1×10^3	$\pm1.4\times10^3$
Mg	4×10^3	6×10^3	5×10^3	$\pm1\times10^3$
Nd	6×10^4	1.05×10^5	1.5×10^5	$\pm9\times10^4$
Pb	882	3.6×10^3	2.21×10^3	$\pm1.36\times10^3$
Pd	14×10^6	28×10^6	21×10^6	$\pm7\times10^6$
Plastics*	750	1.03×10^3	890	±140
Pt	1.9×10^6	5.1×10^6	3.5×10^6	$\pm1.6\times10^6$
Sn	14×10^3	25×10^3	20×10^3	$\pm5.5\times10^3$
Rh	1.3×10^6	3×10^6	2.2×10^6	$\pm0.9\times10^6$
Rubber	160	260	210	±50
W	6×10^3	1.4×10^4	1×10^4	$\pm4\times10^3$
Y/Eu	190	210	200	±10
Zn	1.5×10^3	2.8×10^3	2.2×10^3	±650

Note: Mean is the average of Min and Max. Error = (Max - Min)/2. Data source from London Metal Exchange (http://www.lme.com), and InvestmentMine (http://www.infomine.com); * Source from six-month operation of a recycling plant in China.

Supplementary Table 6 Previous applications of various methods for predicting AM generation

Method	Utilizations
Market supply method	Fluorescent lamp [65]; Refrigerator [66]
Market supply method A	Mobile phone [67]; liquid crystal display [68]
Stanford method	Computer [69]; printer [70]
Consumption and use approach	Mobile phone [67]
Time-step method	Computer [66]; Lithium battery [71]
Stock-based model	Household appliances [72]
Material flow analysis method	WEEE/E-waste [73]

Supplementary Table 7 Data demand's comparison of various methods for AM prediction [67]

	Other designation	Sales (production, import and export)	Lifetime distribution	Possession	Historical collected/ generation amount	Saturation factor	Substitution effect	Influential factors	Econometric indicators
Market supply method	Classic market supply method/sales/simple delay	√	√						
Market supply A method	Distribution delay	√	√						
Stanford method		√	√						
Carnegie Mellon method		√	√						
Consumption and use approach	Approximation/ estimate formula/batch leaching		√	√					
Time-step method		√		√	√				
MFA method		√		√					
ICER model		√				√			
Use-phase analysis			√	√			√		
Time-series model	Projection				√				
Factor model					√			√	
Econometric analysis									√
Direct waste analysis	Waste facility record compilation/disposal related analysis				√				

Supplementary Table 8 w_i: Estimation of average PC weight

**Year**	**2000**	**2001**	**2002**	**2003**	**2004**	**2005**	**2006**	**2007**	**2008**	**2009**	**2010**	**2011**	**2012**	**2013**	**2014**	**2015**	**2016**	**2017**	**2018**	**2019**	**2020**
CRT (%)	95	90	85	80	75	70	65	60	55	50	45	40	35	30	25	20	15	10	5	0	0
LCD (%)	5	10	15	20	25	30	35	40	45	50	55	60	65	70	75	80	85	90	95	100	100
**PC weight (kg)**	**20.1**	**19.8**	**19.5**	**19.1**	**18.8**	**18.5**	**18.2**	**17.8**	**17.5**	**17.2**	**16.8**	**16.5**	**16.2**	**15.9**	**15.5**	**15.2**	**14.9**	**14.5**	**14.2**	**13.9**	**13.9**

Note: The market share of CRT used in PC is gradually shrinking, and CRT will not be utilized for PC in 2019.

Supplementary Table 9 w_i: Estimation of average TV weight

**Year**	**2000**	**2001**	**2002**	**2003**	**2004**	**2005**	**2006**	**2007**	**2008**	**2009**	**2010**
**CRT (%)**	95	90	80	70	60	50	40	30	20	10	0
**LCD (%)**	5	10	20	30	40	50	60	70	80	90	100
**TV weight (kg)**	**27.3**	**26.8**	**25.8**	**24.9**	**23.9**	**22.9**	**21.9**	**20.9**	**20.0**	**19.0**	**18.0**

Note: The market share of CRT used in TV is also gradually shrinking, and CRT will not be utilized for TV in 2010.

Supplementary Table 10 Prohibitive imported directory of solid waste and their implemented time

NO	Solid waste catalog	Implemented time	NO	Solid waste catalog	Implemented time	NO	Solid waste catalog	Implemented time
Ⅰ. Waste plant and animal products			51	Waste acid lotion and waste oil	Dec., 31, 2017	99	Cr scrap	Dec., 31, 2017
1	Waste hair of people	Dec., 31, 2017	52	Organic-containing chemical waste	Dec., 31, 2017	100	Be scrap	Dec., 31, 2017
2	Pig hair scrap	Dec., 31, 2017	53	Other chemical waste	Dec., 31, 2017	101	Cr scrap	Dec., 31, 2017
3	Animal hair scrap	Dec., 31, 2017	54	Other unlisted chemical waste	Dec., 31, 2017	102	Tl scrap	Dec., 31, 2017
4	Feather scrap	Dec., 31, 2017	55	Sludge	Dec., 31, 2017	103	In scrap	Dec., 31, 2017
5	Bone scrap containing ingredients of cattle and sheep	Dec., 31, 2017	56	Medical waste	Dec., 31, 2017	104	Wooden scrap	Dec., 31, 2019
6	Other bone scrap	Dec., 31, 2017	VI. Plastic scrap and heels			105	Soft wood scrap	Dec., 31, 2019
7	Animal tooth scrap	Dec., 31, 2017	57	Ethylene polymer plastic scrap and heels	Dec., 31, 2018	106	Stainless steel scrap	Dec., 31, 2019
8	Waste horse hair	Dec., 31, 2017	58	Aluminum-plastic composite membrane	Dec., 31, 2018	107	W scrap	Dec., 31, 2019
9	Oil tanning reclaimed grease	Dec., 31, 2017	59	Butyl acrylate-methacrylic acid-styrene polymer plastic scrap and heels	Dec., 31, 2018	108	Mg scrap	Dec., 31, 2019
10	Cane Molasses	Dec., 31, 2017	60	Vanadium-containing (20% ≥ V_2O_5>10%) slag ore, ash, and residue	Dec., 31, 2018	109	Bi scrap	Dec., 31, 2019
11	Other molasses	Dec., 31, 2017	61	PET scrap and heels	Dec., 31, 2018	110	Ti scrap	Dec., 31, 2019
II. Slag ore, ash, and residue			62	Waste PET bottle	Dec., 31, 2018	111	Zr scrap	Dec., 31, 2019

12	Slag ore	Dec., 31, 2017	63	Other plastic scrap and heels	Dec., 31, 2018	112	Ge scrap	Dec., 31, 2019
13	Bitumen crushed stone	Dec., 31, 2017	64	Discarded CD-ROM breakage	Dec., 31, 2018	113	V scrap	Dec., 31, 2019
14	Mica scrap	Dec., 31, 2017	Ⅶ. Waste rubber and leather			114	Nb scrap	Dec., 31, 2019
15	Waste Mg brick	Dec., 31, 2017	65	Waste tire and cutting material	Dec., 31, 2017	115	Hf scrap	Dec., 31, 2019
16	Mn-containing slag from iron and steel smelting	Dec., 31, 2017	66	Waste vulcanized rubber	Dec., 31, 2017	116	Ga & Re scrap	Dec., 31, 2019
17	Other slag from iron and steel smelting	Dec., 31, 2017	67	Un-vulcanized rubber scrap and heel	Dec., 31, 2017	117	Granular or powdered tungsten carbide scrap	Dec., 31, 2019
18	Vanadium-containing slag (V_2O_5>20%) from iron and steel smelting	Dec., 31, 2017	68	Waste hard rubber	Dec., 31, 2017	118	Other tungsten carbide scrap	Dec., 31, 2019
19	Other vanadium slag from iron and steel smelting	Dec., 31, 2017	69	Waste leather residue, ash, sludge and powder	Dec., 31, 2017	119	Scrap car press	Dec., 31, 2018
20	Other slag ore, ash, and residue from iron and steel smelting	Dec., 31, 2017	70	Leather heel	Dec., 31, 2017	120	Recycling of scrap metal and electrical appliances mainly to recover iron and steel scrap hardware and electrical appliances	Dec., 31, 2018
21	Hard zinc-containing slag ore, ash, and residue	Dec., 31, 2017	Ⅷ. Waste paper and cardboard			121	Waste motor based on recovery of Cu	Dec., 31, 2018
22	Other zinc-containing slag ore, ash, and residue	Dec., 31, 2017	71	Waste wall paper, wax coated paper, paraffin paper, carbon paper	Dec., 31, 2017	122	Waste wiring based on recovery of Al	Dec., 31, 2018
23	Lead silt containing slag	Dec., 31, 2017	72	Other waste paper	Dec., 31, 2017	123	Hulk	Dec., 31, 2018

24	Other lead-containing slag ore, ash, and residue	Dec., 31, 2017	IX. Waste textile raw materials and products			XII. Waste battery		
25	Copper-containing slag ore, ash, and residue	Dec., 31, 2017	73	The falling hair of other animals	Dec., 31, 2017	124	Scrap battery and waste battery	Dec., 31, 2017
26	Al-containing slag ore, ash, and residue	Dec., 31, 2017	74	Other animal fine wool waste	Dec., 31, 2017	X III. WEEE and its components & parts		
27	As/Hg/Tl-containing slag ore, ash, and residue	Dec., 31, 2017	75	Other animal coarse wool waste	Dec., 31, 2017	125	Waste computer and office WEEE	Feb., 1, 2000
28	Sb/Be/Cd/Cr-containing slag ore, ash, and residue	Dec., 31, 2017	76	Recovered fiber from animal fine/coarse wool	Dec., 31, 2017	126	Home WEEE	Feb., 1, 2000
29	Other W-containing slag ore, ash, and residue	Dec., 31, 2017	77	Waste cotton yarn	Dec., 31, 2017	127	Waste communication equipment	Feb., 1, 2000
30	Vanadium-containing (V_2O_5>20%) slag ore, ash, and residue	Dec., 31, 2017	78	Cotton recovered fiber	Dec., 31, 2017	128	Waste audio-visual products and radio and television equipment and signaling devices	Feb., 1, 2000
31	Cu-containing (>10%) residue from Cu smelting	Dec., 31, 2017	79	Other waste cotton	Dec., 31, 2017	129	Discarded game machine	Feb., 1, 2000
32	Other metals or compound slag ore, ash, and residue	Dec., 31, 2017	80	Synthetic fiber waste	Dec., 31, 2017	130	Discarded lighting equipment	Feb., 1, 2000
33	Ash and residue from MSW incineration	Dec., 31, 2017	81	Man-made fiber waste	Dec., 31, 2017	131	Discarded electronics components	Feb., 1, 2000
34	Seaweed ash or other ash	Dec., 31, 2017	82	Used cloth	Dec., 31, 2017	132	Discarded medical equipment and radiographic application equipment	Feb., 1, 2000
35	Other slag ore and ash	Dec., 31, 2017	83	Woven fabric made of broken fabrics	Dec., 31, 2017	133	Other WEEE	Feb., 1, 2000

36	PCB and PBB-containing waste oil	Dec., 31, 2017	84	Other waste fabrics	Dec., 31, 2017	XIV. Others		
37	Other waste oil	Dec., 31, 2017	85	Other woven fabrics made of textile materials	Dec., 31, 2017	134	Waste gypsum	Dec., 31, 2017
38	Residue of other mineral oil	Dec., 31, 2017	86	Other waste fabrics	Dec., 31, 2017	135	Asbestos	Dec., 31, 2017
39	Granular slag produced by smelting iron and steel (Mn>25%)	Dec., 31, 2018	X. Waste glass			136	Waste ceramic fiber similar to the physical and chemical properties of asbestos	Dec., 31, 2017
40	Oxide skin produced by rolling steel	Dec., 31, 2018	87	Scrap glass	Dec., 31, 2017	137	Used plastic bags, membranes, nets, and used plastic plastic films and used agricultural plastic hoses collected from home or from domestic waste	Dec., 31, 2017
41	Granular slag produced by smelting iron and steel (Fe>80%)	Dec., 31, 2018	XI. Waste of metal and metal compounds			138	Waste fishing net	Dec., 31, 2017
42	Polysilicon scrap (SI>99.9999999%)	Dec., 31, 2017	88	Ash containing silver or silver compounds	Dec., 31, 2017	139	Waste woven bags and waste sacks	Dec., 31, 2017
43	Other silicon scrap (SI≥99.99%)	Dec., 31, 2017	89	Ash containing other precious metals or its compounds	Dec., 31, 2017	140	Waste paint and waste paint	Dec., 31, 2017
IV. Waste drug			90	Scrap containing Au or Au compounds	Dec., 31, 2017	141	Bamboo fiber waste and leftover	Dec., 31, 2017
44	Waste drug	Dec., 31, 2017	91	Scrap containing Ag or Ag compounds	Dec., 31, 2017	142	Finished waste silicon wafer	Dec., 31, 2017

V. Mixed chemical waste			92	Scrap containing other precious metals or their compounds	Dec., 31, 2017	143	Chorionic waste	Dec., 31, 2017
45	Residual alkali solution of wood pulp	Dec., 31, 2017	93	Sedimentary copper	Dec., 31, 2017	144	Sulphur silt	Dec., 31, 2017
46	MSW	Dec., 31, 2017	94	Lead scrap	Dec., 31, 2017	145	Waste phosphor	Dec., 31, 2017
47	Sludge	Dec., 31, 2017	95	Molybdenum scrap	Dec., 31, 2017	146	Nickel-containing slag, ash and residue	Dec., 31, 2017
48	Medical waste	Dec., 31, 2017	96	Cobalt matte scrap	Dec., 31, 2017	147	Vanadium-containing waste catalyst	Dec., 31, 2017
49	Waste organic solvent	Dec., 31, 2017	97	Cd scrap	Dec., 31, 2017	148	Discarded sleepers	Dec., 31, 2017
50	Other waste organic solvent	Dec., 31, 2017	98	Sb scrap	Dec., 31, 2017	149	Other listed waste	Dec., 31, 2017

Note: Data source from MEE (2017, 2018) [74, 75].

Supplementary Table 11 $I(x)$: Estimation and range of imported AM in 2010-2050

a WEEE (kt) [5]

Year	Estimation	Range*	Year	Estimation	Range	Year	Estimation	Range	Year	Estimation	Range
**2010**	877	[600-1500]	**2021**	115	[0-600]	**2031**	0	[0-600]	**2041**	0	[0-600]
**2011**	808	[600-1500]	**2022**	46	[0-600]	**2032**	0	[0-600]	**2042**	0	[0-600]
**2012**	738	[600-1500]	**2023**	0	[0-600]	**2033**	0	[0-600]	**2043**	0	[0-600]
**2013**	669	[600-1500]	**2024**	0	[0-600]	**2034**	0	[0-600]	**2044**	0	[0-600]
**2014**	600	600	**2025**	0	[0-600]	**2035**	0	[0-600]	**2045**	0	[0-600]
**2015**	531	[0-600]	**2026**	0	[0-600]	**2036**	0	[0-600]	**2046**	0	[0-600]
**2016**	462	[0-600]	**2027**	0	[0-600]	**2037**	0	[0-600]	**2047**	0	[0-600]
**2017**	392	[0-600]	**2028**	0	[0-600]	**2038**	0	[0-600]	**2048**	0	[0-600]
**2018**	323	[0-600]	**2029**	0	[0-600]	**2039**	0	[0-600]	**2049**	0	[0-600]
**2019**	254	[0-600]	**2030**	0	[0-600]	**2040**	0	[0-600]	**2050**	0	[0-600]
**2020**	185	[0-600]									

Note: *600 kt was determined from the latest survey in 2015, and 1500 kt was imported WEEE weight in 2001[5].

b Copper scrap (Mt)

Year	Estimation	Range*	Year	Estimation	Range	Year	Estimation	Range	Year	Estimation	Range
**2005**	4.82	4.82	**2017**	3.05	[2.74-3.36]	**2029**	0	[0-0.27]	**2040**	0	[0-0.27]
**2006**	4.94	4.94	**2018**	2.74	[2.19-3.29]	**2030**	0	[0-0.27]	**2041**	0	[0-0.27]
**2007**	5.58	5.58	**2019**	2.43	[1.70-3.16]	**2031**	0	[0-0.27]	**2042**	0	[0-0.27]
**2008**	5.58	5.58	**2020**	2.13	[1.28-2.98]	**2032**	0	[0-0.27]	**2043**	0	[0-0.27]
**2009**	4	4	**2021**	1.82	[0.91-2.73]	**2033**	0	[0-0.27]	**2044**	0	[0-0.27]
**2010**	4.36	4.36	**2022**	1.51	[0.60-2.42]	**2034**	0	[0-0.27]	**2045**	0	[0-0.27]
**2011**	4.69	4.69	**2023**	1.20	[0.36-2.04]	**2035**	0	[0-0.27]	**2046**	0	[0-0.27]
**2012**	4.86	4.86	**2024**	0.89	[0.18-1.60]	**2036**	0	[0-0.27]	**2047**	0	[0-0.27]
**2013**	4.37	4.37	**2025**	0.58	[0.06-1.10]	**2037**	0	[0-0.27]	**2048**	0	[0-0.27]
**2014**	3.87	3.87	**2026**	0.27	[0-0.54]	**2038**	0	[0-0.27]	**2049**	0	[0-0.27]
**2015**	3.66	3.66	**2027**	0	[0-0.27]	**2039**	0	[0-0.27]	**2050**	0	[0-0.27]
**2016**	3.35	3.35	**2028**	0	[0-0.27]						

Note: *Value for 2010-2016 is real data. Range for 2017 is assumed with 10% of estimation for 2017. This rate is given 10% increase owing to growing uncertainty.

Thus, the error rate in 2026 will be 100% so that its range is 0-0.54.

c Aluminum scrap (Mt)

Year	Estimation	Range*	Year	Estimation	Range	Year	Estimation	Range	Year	Estimation	Range
**2005**	1.69	1.69	**2017**	1.79	[1.61-1.99]	**2029**	0	[0-0.37]	**2040**	0	[0-0.37]
**2006**	1.77	1.77	**2018**	1.64	[1.31-1.97]	**2030**	0	[0-0.37]	**2041**	0	[0-0.37]
**2007**	2.09	2.09	**2019**	1.48	[1.04-1.92]	**2031**	0	[0-0.37]	**2042**	0	[0-0.37]
**2008**	2.15	2.15	**2020**	1.32	[0.79-1.85]	**2032**	0	[0-0.37]	**2043**	0	[0-0.37]
**2009**	2.63	2.63	**2021**	1.16	[0.65-1.72]	**2033**	0	[0-0.37]	**2044**	0	[0-0.37]
**2010**	2.85	2.85	**2022**	1.00	[0.40-1.60]	**2034**	0	[0-0.37]	**2045**	0	[0-0.37]
**2011**	2.69	2.69	**2023**	0.84	[0.25-1.43]	**2035**	0	[0-0.37]	**2046**	0	[0-0.37]
**2012**	2.59	2.59	**2024**	0.68	[0.13-1.23]	**2036**	0	[0-0.37]	**2047**	0	[0-0.37]
**2013**	2.50	2.50	**2025**	0.53	[0.06-1.00]	**2037**	0	[0-0.37]	**2048**	0	[0-0.37]
**2014**	2.31	2.31	**2026**	0.37	[0-0.74]	**2038**	0	[0-0.37]	**2049**	0	[0-0.37]
**2015**	2.09	2.09	**2027**	0.21	[0-0.58]	**2039**	0	[0-0.37]	**2050**	0	[0-0.37]
**2016**	1.92	1.92	**2028**	0.05	[0-0.42]						

Note: *Value for 2010-2016 is real data. Range for 2017 is assumed with 10% of estimation for 2017. This rate is given 10% increase owing to growing uncertainty. Thus, the error rate in 2026 will be 100% so that its range is 0-0.74.

d Steel scrap (Mt)

Year	Estimation	Range*	Year	Estimation	Range	Year	Estimation	Range	Year	Estimation	Range
**2007**	3.39	3.39	**2018**	1.74	[1.39-2.09]	**2029**	0	[0-0.13]	**2040**	0	[0-0.13]
**2008**	5.85	5.85	**2019**	1.54	[1.08-2.00]	**2030**	0	[0-0.13]	**2041**	0	[0-0.13]
**2009**	13.69	13.69	**2020**	1.34	[0.80-1.88]	**2031**	0	[0-0.13]	**2042**	0	[0-0.13]
**2010**	5.85	5.85	**2021**	1.14	[0.57-1.71]	**2032**	0	[0-0.13]	**2043**	0	[0-0.13]
**2011**	6.77	6.77	**2022**	0.94	[0.28-1.50]	**2033**	0	[0-0.13]	**2044**	0	[0-0.13]
**2012**	4.97	4.97	**2023**	0.73	[0.22-1.24]	**2034**	0	[0-0.13]	**2045**	0	[0-0.13]
**2013**	4.47	4.47	**2024**	0.53	[0.10-0.96]	**2035**	0	[0-0.13]	**2046**	0	[0-0.13]
**2014**	2.56	2.56	**2025**	0.33	[0.03-0.63]	**2036**	0	[0-0.13]	**2047**	0	[0-0.13]
**2015**	2.33	2.33	**2026**	0.13	[0-0.26]	**2037**	0	[0-0.13]	**2048**	0	[0-0.13]
**2016**	2.16	2.16	**2027**	0	[0-0.13]	**2038**	0	[0-0.13]	**2049**	0	[0-0.13]
**2017**	1.95	[1.76-2.14]	**2028**	0	[0-0.13]	**2039**	0	[0-0.13]	**2050**	0	[0-0.13]

Note: *Value for 2010-2016 is real data. Range for 2017 is assumed with 10% of estimation for 2017. This rate is given 10% increase owing to growing uncertainty.

Thus, the error rate in 2026 will be 100% so that its range is 0-0.26.

e Plastics scrap (Mt)

**Year**	Estimation	Range*	**Year**	Estimation	Range	**Year**	Estimation	Range	**Year**	Estimation	Range
**2010**	8.01	8.01	**2021**	6.02	[3.01-9.03]	**2031**	3.36	[0-8.05]	**2041**	0.69	[0-5.38]
**2011**	8.38	8.38	**2022**	5.76	[2.31-9.21]	**2032**	3.09	[0-7.78]	**2042**	0.42	[0-5.11]
**2012**	8.88	8.88	**2023**	5.49	[1.65-9.22]	**2033**	2.82	[0-7.51]	**2043**	0.16	[0-4.85]
**2013**	7.88	7.88	**2024**	5.22	[1.04-9.40]	**2034**	2.56	[0-7.25]	**2044**	0	[0-4.69]
**2014**	8.26	8.26	**2025**	4.96	[0.50-9.42]	**2035**	2.29	[0-6.98]	**2045**	0	[0-4.69]
**2015**	7.36	7.36	**2026**	4.69	[0-9.38]	**2036**	2.02	[0-6.71]	**2046**	0	[0-4.69]
**2016**	7.35	7.35	**2027**	4.42	[0-9.11]	**2037**	1.76	[0-6.45]	**2047**	0	[0-4.69]
**2017**	7.09	[6.28-7.80]	**2028**	4.16	[0-8.85]	**2038**	1.49	[0-6.18]	**2048**	0	[0-4.69]
**2018**	6.82	[5.46-8.18]	**2029**	3.89	[0-8.55]	**2039**	1.22	[0-5.91]	**2049**	0	[0-4.69]
**2019**	6.56	[4.59-8.53]	**2030**	3.62	[0-8.31]	**2040**	0.96	[0-5.65]	**2050**	0	[0-4.69]
**2020**	6.29	[3.77-8.81]									

Note: *Value for 2010-2016 is real data. Range for 2017 is assumed with 10% of estimation for 2017. This rate is given 10% increase owing to growing uncertainty. Thus, the error rate in 2026 will be 100% so that its range is 0-9.38.

Supplementary Table 12 η, β, and L: Parameters of Weibull lifespan distribution and the regulated maximum lifetime

a EEE [72, 76, 77]

Parameter	Refrigerator	Washing machine	Air conditioner	TV	Desktop PC	Laptop PC	Mobile phone	SMT	Fax machine	Copier	Printer	Monitor	Range hood	Electric water-heater	Gas water-heater
η (scale)	16.5	13.9	12.3	CRT: 12.6<br>FDP: 12	9.6	5.2	7.6	6.5	10.1	10.1	10.1	CRT: 8.5<br>FDP: 7.5	13.5	7.9	7.9
β (shape)	2.6	2.2	2.8	CRT: 2<br>FDP: 2.1	2.1	1.5	1.7	2.1	1.7	1.7	1.7	CRT: 2.2<br>FDP: 2.5	2	1.8	1.8

b Vehicle, bicycle, and cable [78, 79]

**Type**		**η (scale)**	**β (shape)**	**Regulated maximal lifetime for compulsory recycling (y)** [80, 81]
PV	Private passenger vehicle	14.46	4.79	10
	Civil vehicle	13.11	5.33	20
	New-registration civil vehicle	11.53	5.08	20
CT	Heavy duty truck	12.8	5.58	15
	Middle duty truck	10.09	5.58	15
	Small duty truck	8.02	5.58	15
	Mini duty truck	8.02	5.58	12
Car	Taxi	5	10	8
	Private vehicle	14.46	4.79	None
RV		8.02	5.58	15
MC		8.02	5.58	12
Tractor		10 [82]	1.18 [83]	None
Bicycle		6	1.5	None
Wiring & cable [84, 85]		45	2.298	25*

Note: The vehicle made in different countries can be found in China so that their parameters will be referred as the global data. *In 1992-2014, the regulated maximal lifetime for home wiring & cable was 25 years, and since 2015, it was adjusted for over 70 years. The data of η and β is assumed to fit for normal distribution.

Supplementary Table 13 *f*(*x*): Lifetime distribution function of all the relevant products

a EEEs

EEE	η (scale)	β (shape)	*f(x)=* $\frac{\beta}{\eta}(\frac{x}{\eta})^{\beta-1}e^{-(x/\eta)^{\beta}}$
RF	16.5	2.6	$\frac{2.6}{16.5}(\frac{x}{16.5})^{1.6}e^{-(x/16.5)2.6}$
WM	13.9	2.2	$\frac{2.2}{13.9}(\frac{x}{13.9})^{1.2}e^{-(x/13.9)2.2}$
AC	12.3	2.8	$\frac{2.8}{12.3}(\frac{x}{12.3})^{1.8}e^{-(x/12.3)^{2.8}}$
TV-CRT	12.6	2	$\frac{\beta}{12.6}\frac{x}{12.6}e^{-(x/12.6)^{2}}$
TV-FDP	12	2.1	$\frac{2.1}{12}(\frac{x}{12})^{1.1}e^{-(x/12)^{2.1}}$
DPC	9.6	2.1	$\frac{2.1}{9.6}(\frac{x}{9.6})^{1.1}e^{-(x/9.6)^{2.1}}$
LPC	5.2	1.5	$\frac{1.5}{5.2}(\frac{x}{5.2})^{0.5}e^{-(x/5.2)^{1.5}}$
MP	7.6	1.7	$\frac{1.7}{7.6}(\frac{x}{7.6})^{0.7}e^{-(x/7.6)^{1.7}}$
SMT	6.5	2.1	$\frac{2.1}{6.5}(\frac{x}{6.5})^{1.1}e^{-(x/6.5)^{2.1}}$
FM	10.1	1.7	$\frac{1.7}{10.1}(\frac{x}{10.1})^{0.7}e^{-(x/10.1)^{1.7}}$
Copier	10.1	1.7	$\frac{1.7}{10.1}(\frac{x}{10.1})^{0.7}e^{-(x/10.1)^{1.7}}$
Printer	10.1	1.7	$\frac{1.7}{10.1}(\frac{x}{10.1})^{0.7}e^{-(x/10.1)^{1.7}}$
Monitor-CRT	8.5	2.2	$\frac{2.2}{8.5}(\frac{x}{8.5})^{1.2}e^{-(x/8.5)^{2.2}}$
Monitor-FDP	7.5	2.5	$\frac{2.5}{7.5}(\frac{x}{7.5})^{1.5}e^{-(x/7.5)^{2.5}}$
RH	13.5	2	$\frac{2}{13.5}\frac{x}{13.5}e^{-(x/13.5)^{2}}$
EWH	7.9	1.8	$\frac{1.8}{7.9}(\frac{x}{7.9})^{0.8}e^{-(x/\eta)^{1.8}}$
GWH	7.9	1.8	$\frac{1.8}{7.9}(\frac{x}{7.9})^{0.8}e^{-(x/\eta)^{1.8}}$

b Vehicles

Type of vehicle	η (y)	β	Regulated maximization lifetime (y) [80]	Lifetime distribution function

Passenger vehicle	Private passenger vehicle	14.46	4.79	10	$f(x)=\begin{cases}0 & x>10\\ e^{-(x/14.46)^{4.79}} & x=10\\ \frac{4.79}{14.46}(\frac{x}{14.46})^{3.79}e^{-(x/14.46)^{4.79}} & 0\le x<10\\ 0 & x<0\end{cases}$
	Civil vehicle	13.11	5.33	20	$f(x)=\begin{cases}0 & x>20\\ e^{-(x/13.11)^{5.33}} & x=20\\ \frac{5.33}{13.11}(\frac{x}{13.11})^{4.33}e^{-(x/13.11)^{5.33}} & 0\le x<20\\ 0 & x<0\end{cases}$
	New-registration civil vehicle	11.53	5.08	20	$f(x)=\begin{cases}0 & x>20\\ e^{-(x/11.53)^{5.08}} & x=20\\ \frac{5.08}{11.53}(\frac{x}{11.53})^{4.08}e^{-(x/11.53)^{5.08}} & 0\le x<20\\ 0 & x<0\end{cases}$
Cargo truck	Heavy cargo truck	12.8	5.58	15	$f(x)=\begin{cases}0 & x>15\\ e^{-(x/12.8)^{5.58}} & x=15\\ \frac{5.58}{12.8}(\frac{x}{12.8})^{4.58}e^{-(x/12.8)^{5.58}} & 0\le x<15\\ 0 & x<0\end{cases}$
	Medium cargo truck	10.09	5.58	15	$f(x)=\begin{cases}0 & x>15\\ e^{-(x/10.09)^{5.58}} & x=15\\ \frac{5.58}{10.09}(\frac{x}{10.09})^{4.58}e^{-(x/10.09)^{5.58}} & 0\le x<15\\ 0 & x<0\end{cases}$
	Small cargo truck	8.02	5.58	15	$f(x)=\begin{cases}0 & x>15\\ e^{-(x/8.02)^{5.58}} & x=15\\ \frac{5.58}{8.02}(\frac{x}{8.02})^{4.58}e^{-(x/8.02)^{5.58}} & 0\le x<15\\ 0 & x<0\end{cases}$
	Mini cargo truck	8.02	5.58	12	$f(x)=\begin{cases}0 & x>12\\ e^{-(x/8.02)^{5.58}} & x=12\\ \frac{5.58}{8.02}(\frac{x}{8.02})^{4.58}e^{-(x/8.02)^{5.58}} & 0\le x<12\\ 0 & x<0\end{cases}$
Car	Taxi	5	10	8	$f(x)=\begin{cases}0 & x>8\\ e^{-(x/5)^{10}} & x=8\\ \frac{10}{5}(\frac{x}{5})^{9}e^{-(x/5)^{10}} & 0\le x<8\\ 0 & x<0\end{cases}$

	Private vehicle	14.46	4.79	None	$f(x)=\begin{cases}\frac{4.79}{14.46}(\frac{x}{14.46})^{3.79}e^{-(x/14.46)^{4.79}} & x\geq 0\\ 0 & x<0\end{cases}$
Refit vehicle		8.02	5.58	15	$f(x)=\begin{cases}0 & x>15\\ e^{-(x/8.02)^{5.58}} & x=15\\ \frac{5.58}{8.02}(\frac{x}{8.02})^{4.58}e^{-(x/8.02)^{5.58}} & 0\leq x<15\\ 0 & x<0\end{cases}$
Motorcycle		8.02	5.58	12	$f(x)=\begin{cases}0 & x>12\\ e^{-(x/8.02)^{5.58}} & x=12\\ \frac{5.58}{8.02}(\frac{x}{8.02})^{4.58}e^{-(x/8.02)^{5.58}} & 0\leq x<12\\ 0 & x<0\end{cases}$
Tractor		10[82]	1.18[83]	none	$f(x)=\begin{cases}\frac{1.18}{10}(\frac{x}{10})^{0.18}e^{-(x/10)^{1.18}} & x\geq 0\\ 0 & x<0\end{cases}$

Supplementary Table 14 The total estimated consumption of various product from 1990 to 2050

a The estimated unit quantity of EEEs consumption (million)

Year	RF	WM	MP	AC	CRT-TV	FDP-TV	CRT-PC	LCD-PC	SMT	RH	FM	Copier	Printer	LPC	CRT-monitor	LCD-monitor	EWH	GWH	Error (%)
2000	12.79	14.4298	52.479	18.2667	19.019	20.02	6.384	0.336	39.36	3.6615	1.9629	1.5663	17.99109		32.78	1.7255			0
2001	13.5126	13.4161	80.317	23.3364	17.9973	19.997	7.89885	0.87765	40.937	4.5628	3.1819	1.4412	20.63995		34.45	3.8278	1.5331	2.04603	0
2002	15.9887	15.9576	121.4635	31.3511	16.064	20.08	12.43984	2.195265	51.55	4.2646	2.9729	2.0739	20.71047		44.42	7.8383	2.0913	2.51756	0
2003	22.4256	19.6446	182.3137	48.2086	13.1628	18.804	25.7336	6.4334	65.414	4.7725	7.4658	2.6417	30.68809		67.75	16.938	3.9367	3.27306	0
2004	30.0759	25.3341	237.5158	63.9033	7.2048	12.0083	44.81175	14.93725	74.318	6.7744	8.5116	3.2457	49.76988	32.3838	109	36.334	5.3009	5.30268	0
2005	29.8706	30.3552	303.5421	67.6457	9.5	-2.3778	56.59423	24.25467	82.8322	7.301	10.682	5.0608	40.92987	45.6499	73.64	31.559	6.8244	7.02852	0
2006	35.3089	35.605	480.1379	68.4942	10.4	-10.116	60.68686	32.67754	83.754	11.1807	11.886	4.678	46.4027	62.4937	86.84	46.76	10.766	8.23566	0
2007	43.9713	40.051	548.5786	80.1428	10.488	34.9601	72.44028	48.29352	84.7801	12.1688	8.8853	4.5236	42.3469	86.7143	86.63	57.752	15.175	9.71218	0
2008	47.9995	44.47	559.451	81.474	8.2262	41.1314	87.19508	71.34143	91.871	16.3315	7.6991	5.8419	43.3398	108.587	73.51	60.141	17.197	10.871	0
2009	59.3045	49.7363	619.2447	80.7825	4.34979	43.4979	91.07535	91.07535	98.9879	17.1448	6.8351	4.2171	36.4082	150.095	65.62	65.617	14.683	14.2461	0
2010	72.9572	62.4773	998.2736	108.8747	0	51.1003	110.6301	135.2145	118.3003	20.2833	1.8109	5.3466	60.6876	185.841	62.67	76.598	18.728	13.3327	0
2011	86.992	67.1594	1132.577	139.125	0	56.6334	128.1477	192.2216	122.3134	20.3206	2.6813	6.5505	55.1833	238.974	50.72	76.083	24.178	15.688	0
2012	84.27	67.9112	1181.546	132.811	0	66.6952	123.9386	230.1716	128.2352	22.3537	2.6357	6.8701	70.5921	252.894	44.5	82.636	25.126	11.2126	0
2013	92.5574	73.0053	1455.61	130.572	0	67.8821	100.983	235.627	127.7605	25.594	1.7208	6.3538	73.7914	272.789	39.77	92.799	34.926	12.023	0
2014	87.9609	71.144	1712.6	144.633	0	67.316	90.39825	312.32	122.8678	30.8225	1.488	7.1286	66.011	367.89	41	97	34.297	14.7666	0
2015	79.9275	72.745	1969.59	153.582	0	72.9816	78.1238	389.02	116.647	30.4639	1.652	7.342	55.44	312.59	34	103	36.355	15	0
2016	92.383	76.209	2261.087	160.493	0	79.804	62.94675	331.443	120	33.4	1.763	8.1	59.7	290.085	27	109	42	17.7885	10
2017	92	78	2378	167	0	85	45	413	120	35.7	1.61	8.5	59.7	357	19	115	46	19	10
2018	94	80	2592	174	0	89	24	451	120	38.1	1.61	9.0	59.7	377	10	121	50	21	10
2019	95	82	2806	181	0	94	0	489	120	40.5	1.61	9.4	59.7	398	0	128	54	22	10
2020	97	84	3020	188	0	99	0	527	120	42.8	1.61	9.9	59.7	418	0	134	57	24	10
2021	99	86	3234	194	0	103	0	565	120	45.2	1.61	10.3	59.7	439	0	140	61	25	10
2022	101	88	3448	201	0	108	0	603	120	47.6	1.61	10.8	59.7	459	0	146	65	27	10
2023	102	90	3662	208	0	113	0	641	120	49.9	1.61	11.3	59.7	480	0	152	69	29	10
2024	104	92	3876	215	0	118	0	679	120	52.3	1.61	11.7	59.7	500	0	158	73	30	10
2025	106	94	4090	222	0	122	0	717	120	54.7	1.61	12.2	59.7	521	0	164	76	32	10
2026	107	96	4304	229	0	127	0	755	120	57.0	1.61	12.6	59.7	541	0	170	80	34	15
2027	109	98	4518	236	0	132	0	793	120	59.4	1.61	13.1	59.7	562	0	176	84	35	15
2028	111	100	4732	243	0	136	0	831	120	61.8	1.61	13.5	59.7	583	0	182	88	37	15
2029	113	102	4946	250	0	141	0	869	120	64.1	1.61	14.0	59.7	603	0	189	92	38	15
2030	114	104	5160	257	0	146	0	907	120	66.5	1.61	14.4	59.7	624	0	195	96	40	15
2031	116	106	5374	264	0	151	0	945	120	68.9	1.61	14.9	59.7	644	0	201	99	42	20
2032	118	108	5588	271	0	155	0	983	120	71.3	1.61	15.4	59.7	665	0	207	103	43	20

2033	119	110	5803	278	0	160	0	1021	120	73.6	1.61	15.8	59.7	685	0	213	107	45	20
2034	121	112	6017	285	0	165	0	1059	120	76.0	1.61	16.3	59.7	706	0	219	111	46	20
2035	123	114	6231	292	0	169	0	1097	120	78.4	1.61	16.7	59.7	726	0	225	115	48	20
2036	124	116	6445	299	0	174	0	1135	120	80.7	1.61	17.2	59.7	747	0	231	118	50	25
2037	126	118	6659	306	0	179	0	1173	120	83.1	1.61	17.6	59.7	767	0	237	122	51	25
2038	128	120	6873	313	0	184	0	1211	120	85.5	1.61	18.1	59.7	788	0	244	126	53	25
2039	130	122	7087	320	0	188	0	1249	120	87.8	1.61	18.5	59.7	808	0	250	130	55	25
2040	131	124	7301	327	0	193	0	1287	120	90.2	1.61	19.0	59.7	829	0	256	134	56	25
2041	133	126	7515	334	0	198	0	1325	120	92.6	1.61	19.4	59.7	849	0	262	138	58	30
2042	135	128	7729	341	0	202	0	1363	120	94.9	1.61	19.9	59.7	870	0	268	141	59	30
2043	136	130	7943	348	0	207	0	1401	120	97.3	1.61	20.4	59.7	891	0	274	145	61	30
2044	138	132	8157	355	0	212	0	1439	120	99.7	1.61	20.8	59.7	911	0	280	149	63	30
2045	140	134	8371	362	0	217	0	1477	120	102.0	1.61	21.3	59.7	932	0	286	153	64	30
2046	142	136	8585	369	0	221	0	1515	120	104.4	1.61	21.7	59.7	952	0	292	157	66	35
2047	143	138	8799	376	0	226	0	1553	120	106.8	1.61	22.2	59.7	973	0	299	160	67	35
2048	145	140	9013	383	0	231	0	1591	120	109.1	1.61	22.6	59.7	993	0	305	164	69	35
2049	147	142	9227	390	0	236	0	1629	120	111.5	1.61	23.1	59.7	1014	0	311	168	71	35
2050	148	144	9441	397	0	240	0	1667	120	113.9	1.61	23.5	59.7	1034	0	317	172	72	35

Note: basic data is the net production for consumption, and the italic data is the estimated consumption based on the regression.

b The estimated unit quantity of vehicle consumption (million)

Year	Car	PV	CT	RV	MC	Tractor	Bicycle	EV	Error (%)
1990	0.069063					0.039	1.5	0	0
1991	0.135464	0.175742	0.452023			0.045	1.8	0	0
1992	0.190283	0.272582	0.626414			0.05	2	0	0
1993	0.300822	0.292213	0.774868			0.055	2.4864	0	0
1994	0.284976	0.317159	0.785876			0.06	6.2812	0	0
1995	0.404133	0.405454	0.721822			0.063	10.076	0	0
1996	0.449041	0.395192	0.688614			0.084	13.8708	0	0
1997	0.519639	0.435615	0.659318			0.082	17.6656	0	0
1998	0.525149	0.3211	0.661701			0.0678	23.1249	0	0
1999	0.585731	0.4249	0.787636			0.0654	23.9757	0	0
2000	0.628536	0.5779	0.787309			0.041	29.0679	0	0
2001	0.749394	0.721187	0.865947	0.323372	7.084679	0.0382	29.0226	0	0

2002	1.162119	0.897643	1.210221	0.390214	8.548161	0.0454	39.5752	0	0
2003	2.138032	1.150966	1.373725	0.372528	10.40218	0.0488	54.517	0	0
2004	2.419311	1.215549	1.637727	0.499501	10.72398	0.1138	79.0622	0	0
2005	2.813139	1.295721	1.571064	0.501938	8.730347	0.1633	69.0064	0	0
2006	3.887959	1.528759	1.817682	0.562731	12.11988	0.1993	78.8663	0	0
2007	4.748917	1.749189	2.211154	0.743533	15.42128	0.2031	74.752	0	0
2008	4.950539	1.249074	2.392256	0.825816	17.22502	0.2844	63.7487	0	0
2009	7.533599	2.075954	3.29194	0.937568	20.92668	0.3713	57.5765	0	0
2010	9.739603	2.519852	4.501627	1.119563	27.12393	0.3368	68.1948	0	0
2011	10.1757	2.2719	4.167541	*1.10*	19.06777	0.4019	71.6911	0.008368	0
2012	10.71871	2.7175	4.018311	*1.18*	23.17143	0.5273	76.1285	0.012552	0
2013	12.0997	1.6209	4.352868	*1.27*	25.38931	0.6656	75.4525	0.017533	0
2014	12.58018	1.587	4.292219	*1.35*	25.31586	0.6437	79.1014	0.078499	0
2015	11.67536	0.8042	3.340995	*1.44*	26.9905	0.6882	68.82	0.34	0
2016	*12.7*	*1.0*	*4*	*1.53*	*29.6*	0.6184	66.15	0.507	10
2017	*13.3*	*0.8*	*4*	*1.61*	*31.1*	0.3444	71.05	0.777	10
2018	*13.9*	*0.6*	*4*	*1.70*	*32.6*	0.2435	*70*	1.256	10
2019	*14.5*	*0.4*	*4*	*1.78*	*34.2*	*0.31*	*70*	*1.42*	10
2020	*15.1*	*0.4*	*4*	*1.87*	*35.7*	*0.26*	*70*	*1.70*	10
2021	*15.7*	*0.4*	*4*	*1.95*	*37.3*	*0.20*	*70*	*1.98*	10
2022	*16.3*	*0.4*	*4*	*2.04*	*38.8*	*0.15*	*70*	*2.26*	10
2023	*16.9*	*0.4*	*4*	*2.12*	*40.3*	*0.15*	*70*	*2.54*	10
2024	*17.5*	*0.4*	*4*	*2.21*	*41.9*	*0.15*	*70*	*2.82*	10
2025	*18.1*	*0.4*	*4*	*2.29*	*43.4*	*0.15*	*70*	*3.10*	10
2026	*18.7*	*0.4*	*4*	*2.38*	*45.0*	*0.15*	*70*	*3.38*	15
2027	*19.3*	*0.4*	*4*	*2.46*	*46.5*	*0.15*	*70*	*3.66*	15
2028	*19.9*	*0.4*	*4*	*2.55*	*48.0*	*0.15*	*70*	*3.94*	15
2029	*20.5*	*0.4*	*4*	*2.63*	*49.6*	*0.15*	*70*	*4.22*	15
2030	*21.1*	*0.4*	*4*	*2.72*	*51.1*	*0.15*	*70*	*4.49*	15
2031	*21.7*	*0.4*	*4*	*2.80*	*52.7*	*0.15*	*70*	*4.77*	20
2032	*22.3*	*0.4*	*4*	*2.89*	*54.2*	*0.15*	*70*	*5.05*	20
2033	*22.9*	*0.4*	*4*	*2.97*	*55.7*	*0.15*	*70*	*5.33*	20
2034	*23.5*	*0.4*	*4*	*3.06*	*57.3*	*0.15*	*70*	*5.61*	20
2035	*24.1*	*0.4*	*4*	*3.14*	*58.8*	*0.15*	*70*	*5.89*	20

2036	*24.7*	*0.4*	*4*	*3.23*	*60.4*	*0.15*	*70*	*6.17*	25
2037	*25.3*	*0.4*	*4*	*3.31*	*61.9*	*0.15*	*70*	*6.45*	25
2038	*25.9*	*0.4*	*4*	*3.40*	*63.4*	*0.15*	*70*	*6.73*	25
2039	*26.5*	*0.4*	*4*	*3.48*	*65.0*	*0.15*	*70*	*7.01*	25
2040	*27.1*	*0.4*	*4*	*3.57*	*66.5*	*0.15*	*70*	*7.29*	25
2041	*27.7*	*0.4*	*4*	*3.65*	*68.1*	*0.15*	*70*	*7.57*	30
2042	*28.3*	*0.4*	*4*	*3.74*	*69.6*	*0.15*	*70*	*7.84*	30
2043	*28.9*	*0.4*	*4*	*3.83*	*71.1*	*0.15*	*70*	*8.12*	30
2044	*29.5*	*0.4*	*4*	*3.91*	*72.7*	*0.15*	*70*	*8.40*	30
2045	*30.1*	*0.4*	*4*	*4.00*	*74.2*	*0.15*	*70*	*8.68*	30
2046	*30.7*	*0.4*	*4*	*4.08*	*75.8*	*0.15*	*70*	*8.96*	35
2047	*31.3*	*0.4*	*4*	*4.17*	*77.3*	*0.15*	*70*	*9.24*	35
2048	*31.9*	*0.4*	*4*	*4.25*	*78.8*	*0.15*	*70*	*9.52*	35
2049	*32.4*	*0.4*	*4*	*4.34*	*80.4*	*0.15*	*70*	*9.80*	35
2050	*33.0*	*0.4*	*4*	*4.42*	*81.9*	*0.15*	*70*	*10.08*	35

Note: basic data is the net production for consumption, and the italic data is the estimated consumption based on the regression.

c The estimated unit quantity of wiring & cable consumption (million tons)

Year	Power cable	Copper - core aluminum conductors	Telecommunication cable	Electromagnetic wire	Importation	Exportation	Net consumption	Error (%)	WWC importation
1996	*0.4*					0.229648	*0.170352*	0	
1997	*0.5*					0.303428	*0.196572*	0	
1998	*0.6*					0.363381	*0.236619*	0	
1999	*0.7*					0.438151	*0.261849*	0	
2000	*0.9*					0.534069	*0.365931*	0	
2001	1.1074	0.5525			0.2829	0.544667	1.398133	0	
2002	1.27355	0.663			0.316	0.690096	1.562454	0	
2003	1.51225	0.5729			0.3126	0.848022	1.549728	0	
2004	2.34345	0.595			0.3548	1.077072	2.216178	0	
2005	4.0621	0.7934			0.3551	1.234056	3.976544	0	
2006	6.13955	0.926	0.546176		0.3611	1.409157	6.563669	0	
2007	6.8651	1.0776	0.701581		0.3554	1.617206	7.382475	0	
2008	9.821484	0.709248	0.682973		*0.3*	1.69	9.823704	0	
2009	10.69335	0.737902	0.466714	1.15	*0.3*	1.43	11.91796	0	

2010	13.65683	0	0.415386	1.26	0.3	1.74	13.89222	0	2.97
2011	15.39685		0.435415	1.2843	0.3	1.8	15.61657	0	3.0954
2012	20.00985		0.369453	1.3543	0.3	1.86	20.1736	0	3.2076
2013	20.97332		0.346059	1.45	0.3	1.95	21.11938	0	2.8842
2014	27.85184		0.394163	1.55	0.285	2.17	28	0	2.5542
2015	27.1		0.34	1.63	0.3	2.17	27	0	2.41
2016	32		0.32	1.68	0.3	2.27	32	10	2.21
2017	35		0.31	1.76	0.3	2.36	35	10	2.01
2018	38		0.29	1.83	0.3	2.45	38	10	1.81
2019	41		0.27	1.91	0.3	2.54	41	10	1.61
2020	44		0.25	1.98	0.3	2.64	44	10	1.40
2021	47		0.23	2.06	0.3	2.73	47	10	1.20
2022	50		0.21	2.13	0.3	2.82	50	10	1.00
2023	53		0.20	2.21	0.3	2.91	53	10	0.79
2024	56		0.18	2.28	0.3	3.01	56	10	0.59
2025	59		0.16	2.36	0.3	3.10	59	10	0.38
2026	62		0.14	2.44	0.3	3.19	62	15	0.18
2027	65		0.12	2.51	0.3	3.28	65	15	
2028	68		0.11	2.59	0.3	3.37	68	15	
2029	71		0.09	2.66	0.3	3.47	71	15	
2030	74		0.07	2.74	0.3	3.56	74	15	
2031	75		0.05	2.82	0.3	3.65	75	20	
2032	76		0.04	2.89	0.3	3.74	75	20	
2033	77		0.03	2.97	0.3	3.84	76	20	
2034	78		0.02	3.04	0.3	3.93	77	20	
2035	79		0.01	3.12	0.3	4.02	78	20	
2036	80		0	3.19	0.3	4.11	79	25	
2037	81		0	3.27	0.3	4.20	80	25	
2038	82		0	3.34	0.3	4.30	81	25	
2039	83		0	3.42	0.3	4.39	82	25	
2040	84		0	3.50	0.3	4.48	83	25	
2041	85		0	3.57	0.3	4.57	84	30	

2042	*86*		*0*	*3.65*	*0.3*	*4.67*	*85*	*30*	
2043	*87*		*0*	*3.72*	*0.3*	*4.76*	*86*	*30*	
2044	*88*		*0*	*3.80*	*0.3*	*4.85*	*87*	*30*	
2045	*89*		*0*	*3.87*	*0.3*	*4.94*	*88*	*30*	
2046	*90*		*0*	*3.95*	*0.3*	*5.04*	*89*	*35*	
2047	*91*		*0*	*4.02*	*0.3*	*5.13*	*90*	*35*	
2048	*92*		*0*	*4.10*	*0.3*	*5.22*	*91*	*35*	
2049	*93*		*0*	*4.17*	*0.3*	*5.31*	*92*	*35*	
2050	*94*		*0*	*4.25*	*0.3*	*5.40*	*93*	*35*	

d AM importation

Year	WEEE (kt)			Copper scrap (Mt)			Aluminum scrap (Mt)			Iron scrap (Mt)			**Plastics scrap (Mt)**			**Total (Mt)**	
	Estimation	Error	Range*	Estimation	Error	Range	Estimation	Error	Range	Estimation	Error	Range	Estimation	Error	Range	Estimation	Error
2010	*877*	*623*	[600-1500]	4.36	0	4.36	2.85	0	2.85	5.85	0	5.85	8.01	0	8.01	21.947	0.623
2011	*808*	*692*	[600-1500]	4.69	0	4.69	2.69	0	2.69	6.77	0	6.77	8.38	0	8.38	23.338	0.692
2012	*738*	*762*	[600-1500]	4.86	0	4.86	2.59	0	2.59	4.97	0	4.97	8.88	0	8.88	22.038	0.762
2013	*669*	*831*	[600-1500]	4.37	0	4.37	2.5	0	2.5	4.47	0	4.47	7.88	0	7.88	19.889	0.831
2014	600	0	600	3.87	0	3.87	2.31	0	2.31	2.56	0	2.56	8.26	0	8.26	17.6	0
2015	*531*	*531*	[0-600]	3.66	0	3.66	2.09	0	2.09	2.33	0	2.33	7.36	0	7.36	15.971	0.531
2016	*462*	*462*	[0-600]	3.35	0	3.35	1.92	0	1.92	2.16	0	2.16	7.35	0	7.35	15.242	0.462
2017	*392*	*392*	[0-600]	*3.05*	*1.32*	[2.74-3.36]	*1.79*	*0.2*	[1.61-1.99]	*1.95*	*0.19*	[1.76-2.14]	*7.09*	*0.81*	[6.28-7.80]	*14.27*	*2.91*
2018	*323*	*323*	[0-600]	*2.74*	*0.55*	[2.19-3.29]	*1.64*	*0.33*	[1.31-1.97]	*1.74*	*0.35*	[1.39-2.09]	*6.82*	*1.36*	[5.46-8.18]	*13.26*	*2.91*

2019	*254*	*346*	[0-600]	*2.43*	*0.73*	[1.70-3.16]	*1.48*	*0.44*	[1.04-1.92]	*1.54*	*0.46*	[1.08-2.00]	*6.56*	*1.97*	[4.59-8.53]	***12.26***	***3.95***
2020	*185*	*415*	[0-600]	*2.13*	*0.85*	[1.28-2.98]	*1.32*	*0.53*	[0.79-1.85]	*1.34*	*0.54*	[0.80-1.88]	*6.29*	*2.52*	[3.77-8.81]	***11.27***	***4.86***
2021	*115*	*485*	[0-600]	*1.82*	*0.91*	[0.91-2.73]	*1.16*	*0.56*	[0.65-1.72]	*1.14*	*0.57*	[0.57-1.71]	*6.02*	*3.01*	[3.01-9.03]	***10.26***	***5.54***
2022	*46*	*554*	[0-600]	*1.51*	*0.91*	[0.60-2.42]	*1*	*0.6*	[0.40-1.60]	*0.94*	*0.68*	[0.28-1.50]	*5.76*	*3.45*	[2.31-9.21]	***9.26***	***6.19***
2023	*0*	*600*	[0-600]	*1.2*	*0.84*	[0.36-2.04]	*0.84*	*0.59*	[0.25-1.43]	*0.73*	*0.71*	[0.22-1.24]	*5.49*	*3.73*	[1.65-9.22]	***8.26***	***6.47***
2024	*0*	*600*	[0-600]	*0.89*	*0.71*	[0.18-1.60]	*0.68*	*0.55*	[0.13-1.23]	*0.53*	*0.43*	[0.10-0.96]	*5.22*	*4.18*	[1.04-9.40]	***7.32***	***6.47***
2025	*0*	*600*	[0-600]	*0.58*	*0.52*	[0.06-1.10]	*0.53*	*0.47*	[0.06-1.00]	*0.33*	*0.3*	[0.03-0.63]	*4.96*	*4.46*	[0.50-9.42]	***6.4***	***6.35***
2026	*0*	*600*	[0-600]	*0.27*	*0.27*	[0-0.54]	*0.37*	*0.37*	[0-0.74]	*0.13*	*0.13*	[0-0.26]	*4.69*	*4.69*	[0-9.38]	***5.46***	***6.06***
2027	*0*	*600*	[0-600]	*0*	*0.27*	[0-0.27]	*0.21*	*0.37*	[0-0.58]	*0*	*0.13*	[0-0.13]	*4.42*	*4.69*	[0-9.11]	***4.63***	***6.06***
2028	*0*	*600*	[0-600]	*0*	*0.27*	[0-0.27]	*0.05*	*0.37*	[0-0.42]	*0*	*0.13*	[0-0.13]	*4.16*	*4.69*	[0-8.85]	***4.21***	***6.06***
2029	*0*	*600*	[0-600]	*0*	*0.27*	[0-0.27]	*0*	*0.37*	[0-0.37]	*0*	*0.13*	[0-0.13]	*3.89*	*4.69*	[0-8.55]	***3.89***	***6.06***
2030	*0*	*600*	[0-600]	*0*	*0.27*	[0-0.27]	*0*	*0.37*	[0-0.37]	*0*	*0.13*	[0-0.13]	*3.62*	*4.69*	[0-8.31]	***3.62***	***6.06***
2031	*0*	*600*	[0-600]	*0*	*0.27*	[0-0.27]	*0*	*0.37*	[0-0.37]	*0*	*0.13*	[0-0.13]	*3.36*	*4.69*	[0-8.05]	***3.36***	***6.06***
2032	*0*	*600*	[0-600]	*0*	*0.27*	[0-0.27]	*0*	*0.37*	[0-0.37]	*0*	*0.13*	[0-0.13]	*3.09*	*4.69*	[0-7.78]	***3.09***	***6.06***
2033	*0*	*600*	[0-600]	*0*	*0.27*	[0-0.27]	*0*	*0.37*	[0-0.37]	*0*	*0.13*	[0-0.13]	*2.82*	*4.69*	[0-7.51]	***2.82***	***6.06***
2034	*0*	*600*	[0-600]	*0*	*0.27*	[0-0.27]	*0*	*0.37*	[0-0.37]	*0*	*0.13*	[0-0.13]	*2.56*	*4.69*	[0-7.25]	***2.56***	***6.06***

2035	0	600	[0-600]	0	0.27	[0-0.27]	0	0.37	[0-0.37]	0	0.13	[0-0.13]	2.29	4.69	[0-6.98]	2.29	6.06
2036	0	600	[0-600]	0	0.27	[0-0.27]	0	0.37	[0-0.37]	0	0.13	[0-0.13]	2.02	4.69	[0-6.71]	2.02	6.06
2037	0	600	[0-600]	0	0.27	[0-0.27]	0	0.37	[0-0.37]	0	0.13	[0-0.13]	1.76	4.69	[0-6.45]	1.76	6.06
2038	0	600	[0-600]	0	0.27	[0-0.27]	0	0.37	[0-0.37]	0	0.13	[0-0.13]	1.49	4.69	[0-6.18]	1.49	6.06
2039	0	600	[0-600]	0	0.27	[0-0.27]	0	0.37	[0-0.37]	0	0.13	[0-0.13]	1.22	4.69	[0-5.91]	1.22	6.06
2040	0	600	[0-600]	0	0.27	[0-0.27]	0	0.37	[0-0.37]	0	0.13	[0-0.13]	0.96	4.69	[0-5.65]	0.96	6.06
2041	0	600	[0-600]	0	0.27	[0-0.27]	0	0.37	[0-0.37]	0	0.13	[0-0.13]	0.69	4.69	[0-5.38]	0.69	6.06
2042	0	600	[0-600]	0	0.27	[0-0.27]	0	0.37	[0-0.37]	0	0.13	[0-0.13]	0.42	4.69	[0-5.11]	0.42	6.06
2043	0	600	[0-600]	0	0.27	[0-0.27]	0	0.37	[0-0.37]	0	0.13	[0-0.13]	0.16	4.69	[0-4.85]	0.16	6.06
2044	0	600	[0-600]	0	0.27	[0-0.27]	0	0.37	[0-0.37]	0	0.13	[0-0.13]	0	4.69	[0-4.69]	0	6.06
2045	0	600	[0-600]	0	0.27	[0-0.27]	0	0.37	[0-0.37]	0	0.13	[0-0.13]	0	4.69	[0-4.69]	0	6.06
2046	0	600	[0-600]	0	0.27	[0-0.27]	0	0.37	[0-0.37]	0	0.13	[0-0.13]	0	4.69	[0-4.69]	0	6.06
2047	0	600	[0-600]	0	0.27	[0-0.27]	0	0.37	[0-0.37]	0	0.13	[0-0.13]	0	4.69	[0-4.69]	0	6.06
2048	0	600	[0-600]	0	0.27	[0-0.27]	0	0.37	[0-0.37]	0	0.13	[0-0.13]	0	4.69	[0-4.69]	0	6.06
2049	0	600	[0-600]	0	0.27	[0-0.27]	0	0.37	[0-0.37]	0	0.13	[0-0.13]	0	4.69	[0-4.69]	0	6.06
2050	0	600	[0-600]	0	0.27	[0-0.27]	0	0.37	[0-0.37]	0	0.13	[0-0.13]	0	4.69	[0-4.69]	0	6.06

Supplementary Note 1: Terminologies definition and boundary

(1) Solid waste, anthropogenic mineral, and natural mineral [86]

Solid waste is any garbage or refuse, sludge from a wastewater treatment plant, water supply treatment plant, or air pollution control facility and other discarded material, resulting from industrial, commercial, mining, and agricultural operations, and from community activities. For instance, municipal solid waste, e-waste, kitchen waste, metal scrap, paper waste, hazardous waste, and animal manure are the typical solid waste.

Anthropogenic mineral (AM), or called urban mineral, belong to solid waste, is the recycled resources characterizing the product with the orderly physical structure and fixed shape under the condition of human activity. In this study, it is defined as the three group of waste products, i.e., waste electrical and electronic equipment, end of life vehicle, and waste wiring & cable.

Natural mineral is the underground solid resource generated by the naturally occurring process. Kaolinite, pyrite, chalcopyrite, and coal are the typical natural mineral.

(2) New scrap and old scrap[87, 88]

New scrap (or pre-consumer scrap) is the solid waste originated from the fabrication or manufacturing process. For instance, leftover material, smelting offal, industrial sludge are the typical cases.

Old scrap (or postconsumer scrap) is the solid waste generated from end of life product after consumption or utilization, including the anthropogenic mineral. Waste electrical and electronic equipment, end of life vehicle, waste wiring & cable, spent aircraft, scrap-metal goods, and abandoned buildings belong to old scrap.

(3) Primary resources and secondary resources

Primary resources called natural resources (mineral), are resources that exist without actions of humankind. For instance, oil, iron ore, timber, and coal are the primary resources.

Secondary resources are the resources by anthropogenic activity, which contain some valuable materials like metal within them. For instance, copper scrap, packaging waste, end of life vehicle, and waste oil are the typical cases.

(4) Product, resource, and material[89]

Product is a good consisting of a bundle of tangible and intangible attributes that satisfies consumers.

Resource stands for the useful matters, consisting of valuable material.

Material is the element, constituent, or substance of which something is composed or can be made.

(5) Urban mining and virgin mining[90]

Urban mining is the process recycling resources from anthropogenic mineral, consisting of dismantling, crushing, separation, and deep recovery using hydrometallurgy, pyrometallurgy, or biological treatment.

Virgin mining is the process to extract resources from natural mineral, consisting of crushing, separation, and deep recovery using hydrometallurgy, pyrometallurgy, or biological treatment.

(6) Flow, stock, in-use stock, and net addition to in-use stock (Supplementary Fig. 1)[43, 89]

A flow is the change of a stock over time. The yearly-generated anthropogenic mineral belongs to the flow.

A stock is the reservoir that is accumulated over time by inflows and/or depleted by outflows, which is the totally cumulative quantity.

In-use stock is the cumulative product or resource, which is used or consumed functionally.

Net addition to in-use stock in every year is the difference between the yearly demand and yearly-generated anthropogenic mineral, which is the annual theoretical consumption.

Supplementary Note 2: The abbreviations and acronyms of main vocabulary

AC	air conditioner	MCar	medium car
Al	aluminum	MCT	medium cargo truck
Ag	silver	MFA	material flow analysis
AM	anthropogenic mineral	Mg	magnesium
Au	gold	MiCT	mini cargo truck
CDF	cumulative distribution function	MP	mobile phone
Co	cobalt	Mt	million ton
CT	cargo truck	Nd	neodymium
Cu	copper	NPV	New-registration passenger vehicle
DPC	desktop personal computer	Pb	lead
Dy	dysprosium	PC	personal computer
EEE	electrical and electronic equipment	PCV	passenger civil vehicle
ELV	end-of-life vehicle	Pd	palladium
EoL	end of life	PDF	probability density function
Eq.	equation	PPV	private passenger vehicle
Eu	europium	Pt	platinum
EU	European Union	PV	passenger vehicle
EV	electric vehicle	PVC	Private vehicle for civil purpose
e-waste	electrical and electronic waste	RE	rare earth
EWH	electric water heater	RF	refrigerator
Fe	iron	Rh	rhodium
F-C-P	fax machine, copier, and printer	RH	range hood
FM	fax machine	RV	refit vehicle
GWH	gas water heater	SCar	small car
HCT	heavy cargo truck	SCT	small cargo truck
ICT	information and communications technology	SMT	single-machine telephone
In	indium	Sn	tin
La	lanthanum	TV	television
LCar	large car	U.S.	United States
LPC	laptop personal computer	W	tungsten
kg	kilogram	WEEE	waste electrical and electronic equipment
kt	kiloton	WM	washing machine
M	million	WWC	waste wiring and cable
M-CRT	CRT monitor used for mainframe	Y	yttrium
M-FDP	FDP monitor used for mainframe	Zn	zinc
MC	motorcycle		

Supplementary Note 3: Available data pre-mining for estimation

Regarding all the related products, we identified the scale and shape parameters in the Weibull distribution function, depicted in detail in Supplementary Table 13. Their annual failure rates with probability density function are shown in Supplementary Fig. 6. The two zones of in-use and waste are located above and below the function curve, respectively. It can be utilized to determine the yearly obsolete amount and stocked resources of various WEEE, ELV, and WWC.

According to the net production (demand) of various products, obtained from Supplementary Tables 1 and 2, regressions for the recent years indicate that the growth of most net production maintains an excellent linear regression, with a correlation rate of over 0.90 (Supplementary Figs. 7-9). Since there has been a distinct fluctuation in the net production of SMT, FM, printer, passenger vehicle, cargo truck, and bicycle, the fixed values of 120-, 1.61-, 59.73-, 0.4-, 4-, and 70- million were assumed for future annual production, respectively. The total estimated consumption of products from 1990s to 2050 is given in Supplementary excel table.

Supplementary Note 4: Comparison of this study to other studies and real-world data

Regarding five typical types of WEEE (e.g., RF, WM, AC, TV, and PC), their quantity in the year of 2010-2016 without importation in this study is lower than the values from others previous studies (Supplementary Fig. 13). The difference can be attributed to two points of the previous works: old used data sources before 2010 and different forecasting methods. Moreover, in weight, the gaps among these studies are not significant. The bigger gap is from the result indicated by Duan et al. (2016), which is higher than this study because more types of WEEE were considered in Duan et al. (2016). Regarding typical ELV quantity, some previous works indicate no distinct difference to this study. The biggest difference is given from Xi et al. (2014) only using the simple linear regression. Additionally, the registered vehicle has been estimated in this study for further comparison with real-world data. They demonstrate the almost value without significant difference (Supplementary Fig. 14). All the discussions can verify and validate the above results of this study, and further consolidate the relevant results.

Supplementary References

1. Ambler T, Witzel M, Xi C. *Doing business in China*, Fifth edn. GMB Publishing Ltd. (2006).

2. Koetse M. Mirror of Time: Chinese Weddings Through the Decades.). What's on Weibo (2017).

3. Wen H. Dazzling the eyes: television and the modernization ideal in 1980s China.). University of Iowa (2009).

4. BBC. 40 years of reform and opening up: China's home 'Four Fashionable Items'.) (2018).

5. Zeng X, Gong R, Chen W-Q, Li J. Uncovering the Recycling Potential of "New" WEEE in China. *Environmental Science & Technology* **50**, 1347-1358 (2016).

6. Arrow K*, et al.* Economic Growth, Carrying Capacity, and the Environment. *Science* **268**, 520-521 (1995).

7. Genieys S, Volpert V, Auger P. Pattern and Waves for a Model in Population Dynamics with Nonlocal Consumption of Resources. *Mathematical Modelling of Natural Phenomena* **1**, 63-80 (2006).

8. Yang JX, Lu B, Xu C. WEEE flow and mitigating measures in China. *Waste Management* **28**, 1589-1597 (2008).

9. Li J, Tian B, Liu T, Liu H, Wen X, Honda Si. Status quo of e-waste management in mainland China. *J Mater Cycles Waste Manag* **8**, 13-20 (2006).

10. Liu X, Tanaka M, Matsui Y. Generation amount prediction and material flow analysis of electronic waste: a case study in Beijing, China. *Waste Manage Res* **24**, 434-445 (2006).

11. Veenstra A, Wang C, Fan W, Ru Y. An analysis of E-waste flows in China. *The International Journal of Advanced Manufacturing Technology* **47**, 449-459 (2010).

12. Baldé CP, Wang F, Kuehr R, Huisman J. *The global e-waste monitor -2014*. United Nations University, IAS-SCYCLE (2015).

13. Baldé CP, Forti V, Gray V, Kuehr R, Stegmann P. *The Global E-waste Monitor 2017: Quantities, Flows, and Resources*. International Telecommunication Union (ITU) & International Solid Waste Association (ISWA) (2017).

14. Duan H, Hu J, Tan Q, Liu L, Wang Y, Li J. Systematic characterization of generation and management of e-waste in China. *Environ Sci Pollut Res* **23**, 1929-1943 (2016).

15. Xi H, Yang Y, Chen Y. Forcasting the near-future obsolescence, increase, and stock of vehicle in China. *Highways & Automotive Applications*, 30-34 (2014).

16. Xue J, Hu S, Yang Q. Potentials of the Renewable Resource of Scrapped Cars in China. *China Population Resources and Environment* **23**, 169-176 (2013).

17. Jin X, Chu J, Cui P, Zhang T, Tian G, Wang H. Analysis on Modeling of Utilization System of Automobile Renewable Resources. *Systems Engineering* **26**, 69-73 (2008).

18. CHEARI. White Book on WEEE Recycling Industry in China in 2014.). China Household Electric Appliance Research Institute (2015).

19. CHEARI. White Book on WEEE Recycling Industry in China in 2015.). China Household Electric Appliance Research Institute (2016).

20. CHEARI. White Book on WEEE Recycling Industry in China in 2016.). China Household Electric Appliance Research Institute (2017).

21. CHEARI. White Book on WEEE Recycling Industry in China in 2017.). China Household Electric Appliance Research Institute (2018).

22. CHEARI. White Book on WEEE Recycling Industry in China in 2018.). China Household Electric Appliance Research Institute (2019).

23. Cabrera Serrenho A, Allwood JM. Material Stock Demographics: Cars in Great Britain. *Environmental Science & Technology* **50**, 3002-3009 (2016).

24. Lewis GM*, et al.* Green Principles for Vehicle Lightweighting. *Environmental Science & Technology* **53**, 4063-4077 (2019).

25. Cucchiella F, D'Adamo I, Lenny Koh SC, Rosa P. Recycling of WEEEs: An economic assessment of present and future e-waste streams. *Renewable and Sustainable Energy Reviews* **51**, 263-272 (2015).

26. Zeng X. Mechanism and Technology of Nonferrous Metals Recycling from Typical E-waste Parts. In: *School of Environment*). Tsinghua University (2014).

27. Reuter M*, et al.* Metal recycling: opportunities, limits, infrastructure, A Report of the Working Group on the Global Metal Flows to the International Resource Panel. (ed International Resource Panel WGotGMF). UNEP (2013).

28. Shen X, Li L, Wu X, Wang P, Wang L. Recovery of rare earth from waste CRT phosphor. *CIESC Journal* **66**, 1498-1504 (2015).

29. Zeng X, Li J. Spent rechargeable lithium batteries in e-waste: composition and its implications. *Front Env Sci Eng* **8**, 792-796 (2014).

30. Yan G, Xue M, Xu Z. Disposal of waste computer hard disk drive: data destruction and resources recycling. *Waste Manage Res* **31**, 559-567 (2013).

31. Zepf V. *Rare Earth Elements. A New Approach to the Nexus of Supply, Demand and Use: Exemplified along the Use of Neodymium in Permanent Magnets*. Springer Berlin Heidelberg (2013).

32. Zeng X, Wang F, Sun X, Li J. Recycling Indium from Scraped Glass of Liquid Crystal Display: Process Optimizing and Mechanism Exploring. *Acs Sustain Chem Eng* **3**, 1306-1312 (2015).

33. Manhart A, Schleicher T, Degreif S. Global circular economy of strategic metals-the Best-of-two-Worlds Approach (Bo2W).). Oeko Institute (2014).

34. Bandara HMD, Darcy JW, Apelian D, Emmert MH. Value Analysis of Neodymium Content in Shredder Feed: Toward Enabling the Feasibility of Rare Earth Magnet Recycling. *Environmental Science & Technology* **48**, 6553-6560 (2014).

35. Peeters JR*, et al.* Forecasting waste compositions: A case study on plastic waste of electronic display housings. *Waste Management* **46**, 28-39 (2015).

36. Zeng X, Li J. Measuring the recyclability of e-waste: an innovative method and its implications. *Journal of Cleaner Production* **131**, 156-162 (2016).

37. Chen Y, Chen M, Li Y, Wang B, Chen S, Xu Z. Impact of technological innovation and regulation development on e-waste toxicity: a case study of waste mobile phones. *Sci Rep* **8**, 7100 (2018).

38. Singh N, Duan H, Yin F, Song Q, Li J. Characterizing the Materials Composition and Recovery Potential from Waste Mobile Phones: A Comparative Evaluation of Cellular and Smart Phones. *Acs Sustain Chem Eng* **6**, 13016-13024 (2018).

39. Christian B, Romanov A, Romanova I, Turbini L. Elemental Compositions of Over 80 Cell Phones. *Journal of Elec Materi* **43**, 4199-4213 (2014).

40. Sahan M, Kucuker MA, Demirel B, Kuchta K, Hursthouse A. Determination of Metal Content of Waste Mobile Phones and Estimation of Their Recovery Potential in Turkey. *Int J Env Res Pub He* **16**, 887 (2019).

41. Li W, Xia K, Gao L, Chao K-M. Selective disassembly planning for waste electrical and electronic equipment with case studies on liquid crystaldisplays. *Robot Cim-Int Manuf* **29**, 248-260 (2013).

42. Thavalingam V, Karunasena G. Mobile phone waste management in developing countries: A case of Sri Lanka. *Resour Conserv Recy* **109**, 34-43 (2016).

43. Kuong IH, Li J, Zhang J, Zeng X. Estimating the Evolution of Urban Mining Resources in Hong Kong, up to the Year 2050. *Environmental Science & Technology* **53**, 1394-1403 (2019).

44. Yang C, Tan Q, Zeng X, Zhang Y, Wang Z, Li J. Measuring the sustainability of tin in China. *Science of The Total Environment* **635**, 1351-1359 (2018).

45. Innocenzi V, De Michelis I, Ferella F, Beolchini F, Kopacek B, Vegliò F. Recovery of yttrium from fluorescent powder of cathode ray tube, CRT: Zn removal by sulphide precipitation. *Waste Management* **33**, 2364-2371 (2013).

46. Wu Y, Jia P, Wang W, Wang B, Zhang Q. Leaching of rare earth elements from waste fluorescent power of cathode ray tube by molten salts. *Journal of The Chinese Society of Rare Earths* **31**, 723-728 (2013).

47. Shen X, Li L, Wu X, Wang P, Wang L. Recovery of rare earth from waste CRT phosphor. *CIESC Journal* **66**, 1498-1505 (2015).

48. Staudinger J, Keoleian GA. Management of End-of Life Vehicles (ELVs) in the US.) (2001).

49. Vermeulen I, Van Caneghem J, Block C, Baeyens J, Vandecasteele C. Automotive shredder residue (ASR): Reviewing its production from end-of-life vehicles (ELVs) and its recycling, energy or

chemicals' valorisation. *Journal of Hazardous Materials* **190**, 8-27 (2011).

50. Kanari N, Pineau J-L, Shallari S. End-of-life vehicle recycling in the european union. *JOM* **55**, 15-19 (2003).

51. Blount GN. End-of-life vehicles recovery: process description, its impact and direction of research. *J Mekanikal* **21**, 40-52 (2006).

52. Jody BJ, Daniels EJ, Duranceau CM, Pomykala JA, Spangenberger JS. End-of-life vehicle recycling : state of the art of resource recovery from shredder residue.). Argonne National Laboratory (2011).

53. Ferrão P, Nazareth P, Amaral J. Strategies for Meeting EU End-of-Life Vehicle Reuse/Recovery Targets. *Journal of Industrial Ecology* **10**, 77-93 (2006).

54. Sun J, Liu F, Chai J. Current status of compositions of end-of-life vehicle and their recycling in China. *Shanghai Auto*, 54-58 (2014).

55. Widmer R, Du X, Haag O, Restrepo E, Wäger PA. Scarce Metals in Conventional Passenger Vehicles and End-of-Life Vehicle Shredder Output. *Environmental Science & Technology* **49**, 4591-4599 (2015).

56. Restrepo E, Løvik AN, Widmer R, Wäger P, Müller DB. Historical Penetration Patterns of Automobile Electronic Control Systems and Implications for Critical Raw Materials Recycling. *Resources* **8**, 58 (2019).

57. Horton PM, Allwood JM. Yield improvement opportunities for manufacturing automotive sheet metal components. *J Mater Process Tech* **249**, 78-88 (2017).

58. Cheah L, Heywood J, Kirchain R. Aluminum Stock and Flows in U.S. Passenger Vehicles and Implications for Energy Use. *Journal of Industrial Ecology* **13**, 718-734 (2009).

59. Harper E, Graedel T. Illuminating tungsten's life cycle in the United States: 1975 – 2000. *Environmental Science & Technology* **42**, 3835-3842 (2008).

60. Cao F, Yang H, Wang W, Zhang L. General Situation and Analysis of Supply and Demand of Global Tungsten Resources. *Conservation and Utilization of Mineral Resources*, 145-150 (2018).

61. Hao H*, et al.* Securing Platinum-Group Metals for Transport Low-Carbon Transition. *One Earth* **1**, 117-125 (2019).

62. Restrepo E, Løvik AN, Wäger P, Widmer R, Lonka R, Müller DB. Stocks, Flows, and Distribution of Critical Metals in Embedded Electronics in Passenger Vehicles. *Environmental Science & Technology* **51**, 1129-1139 (2017).

63. Huang C. On the direct use of recovered scrap copper and saving copper with aluminum in the Chinese cable industry. *Engineering Sciences*, 2-11 (2008).

64. Huang C. On the copper saving with aluminium in the Chinese cable industry. *Electric Wire & Cable*, 1-3 (2008).

65. Tan Q, Li J. A study of waste fluorescent lamp generation in mainland China. *Journal of Cleaner*

Production **81**, 227-233 (2014).

66. Araújo MG, Magrini A, Mahler CF, Bilitewski B. A model for estimation of potential generation of waste electrical and electronic equipment in Brazil. *Waste Management* **32**, 335-342 (2012).

67. Li B, Yang J, Lu B, Song X. Estimation of retired mobile phones generation in China: A comparative study on methodology. *Waste Management* **35**, 247-254 (2014).

68. Liu Z, Xu Z, Huang H, Li B. A study of waste liquid crystal display generation in mainland China. *Waste Manage Res* **34**, 58-66 (2016).

69. Mmereki D, Li B, Wang L. Estimation of waste electronic and electrical equipment arising in Botswana-A case study of Gaborone City. *International Journal of Environmental Sciences* **3**, 441-452 (2012).

70. Song Q, *et al.* Measuring the generation and management status of waste office equipment in China: a case study of waste printers. *Journal of Cleaner Production* **112, Part 5**, 4461-4468 (2016).

71. Zeng X, Li J, Ren Y. Prediction of Various Discarded Lithium Batteries in China. In: *2012 IEEE International Symposium on Sustainable Systems and Technology (ISSST)*). IEEE (2012).

72. Zhang L, Yuan Z, Bi J, Huang L. Estimating future generation of obsolete household appliances in China. *Waste Manage Res* **30**, 1160-1168 (2012).

73. Yedla S. Development of a methodology for electronic waste estimation: A material flow analysis-based SYE-Waste Model. *Waste Manage Res* **34**, 81-86 (2016).

74. MEE. Notice on Release of Imported Waste Management Catalog (2017).). General Office of MEP (2017).

75. MEE. Bulletin on Adjusting Management Directory of Imported Waste.). Ministry of Ecology and Environment (2018).

76. Baldé CP*, et al. E-waste statistics: Guidelines on classifications, reporting and indicators*. United Nations University, IAS - SCYCLE (2015).

77. Wang F, Huisman J, Stevels A, Baldé CP. Enhancing e-waste estimates: Improving data quality by multivariate Input–Output Analysis. *Waste Management* **33**, 2397-2407 (2013).

78. Yano J, Muroi T, Sakai S-i. Rare earth element recovery potentials from end-of-life hybrid electric vehicle components in 2010–2030. *J Mater Cycles Waste Manag*, 1-10 (2015).

79. Hao H, Wang H, Ouyang M, Cheng F. Vehicle survival patterns in China. *Science China Technological Sciences* **54**, 625-629 (2011).

80. Ministry-of-Commerce. Provisions on the Standards for Compulsory Retirement of Motor Vehicles.). Department of Treaty and Law, Ministry of Commerce (2012).

81. Chen M, Zhang F. End-of-Life vehicle recovery in china: Consideration and innovation following the EU ELV directive. *JOM* **61**, 45-52 (2009).

82. Wei J, Zheng X. Fatigure life prediction of shaft of tractor under original load spectrum. *Acta Mechanica Solida Sinica* **21**, 175-178 (2000).

83. Duan T. An investigation and analysis on the failure rate of tractors. *Acta Agriculturae Universitatis Henanensis* **20**, 11-22 (1986).

84. Xu T, Li M, Liu y, Zhou W, Xu Y, Zhou C. High-voltage cable life assessment based on zero-failure data parameter estimation of Weibull distribution. *Engineering Journal of Wuhan University* **50**, (2017).

85. Zhang T, Wang C, Sun Q, Zhang H. The estimation for state of cable based on improved Weibull distribution model. *China Engineering Sciences* **10**, 42-46 (2008).

86. Zeng X, Li J. Urban Mining and Its Resources Adjustment: Characteristics, Sustainability, and Extraction. *SCIENTIA SINICA Terrae* **48**, 288-298 (2018).

87. Graedel T*, et al.* What Do We Know About Metal Recycling Rates? *Journal of Industrial Ecology* **15**, 355-366 (2011).

88. Tilton JE. *World metal demand: trends and prospects*. Routledge (2015).

89. Brunner PH, Rechberger H. *Handbook of Material Flow Analysis: For Environmental, Resource, and Waste Engineers*, Second edn. CRC Press (2016).

90. Zeng X, Mathews JA, Li J. Urban Mining of E-Waste is Becoming More Cost-Effective Than Virgin Mining. *Environmental Science & Technology* **52**, 4835-4841 (2018).
