# Supplementary material for: Mapping anthropogenic mineral generation in China and its implications for a circular economy
Source: Nat Commun. 2020 Mar 25;11:1544. doi: 10.1038/s41467-020-15246-4 (PMC7096490; doi:10.1038/s41467-020-15246-4)
Supplement: Supplementary file 3 — Reporting Summary [file 41467_2020_15246_MOESM3_ESM.pdf]

nature research

Corresponding author(s): Jinhui Li

Last updated by author(s): Feb 24, 2020

Reporting Summary

Nature Research wishes to improve the reproducibility of the work that we publish. This form provides structure for consistency and transparency in reporting. For further information on Nature Research policies, see Authors & Referees and the Editorial Policy Checklist.

Statistics

For all statistical analyses, confirm that the following items are present in the figure legend, table legend, main text, or Methods section.

n/a	Confirmed	
☒	☐	The exact sample size (*n*) for each experimental group/condition, given as a discrete number and unit of measurement
☒	☐	A statement on whether measurements were taken from distinct samples or whether the same sample was measured repeatedly
☒	☐	The statistical test(s) used AND whether they are one- or two-sided<br>*Only common tests should be described solely by name; describe more complex techniques in the Methods section.*
☒	☐	A description of all covariates tested
☒	☐	A description of any assumptions or corrections, such as tests of normality and adjustment for multiple comparisons
☐	☒	A full description of the statistical parameters including central tendency (e.g. means) or other basic estimates (e.g. regression coefficient) AND variation (e.g. standard deviation) or associated estimates of uncertainty (e.g. confidence intervals)
☐	☒	For null hypothesis testing, the test statistic (e.g. *F*, *t*, *r*) with confidence intervals, effect sizes, degrees of freedom and *P* value noted<br>*Give P values as exact values whenever suitable.*
☒	☐	For Bayesian analysis, information on the choice of priors and Markov chain Monte Carlo settings
☒	☐	For hierarchical and complex designs, identification of the appropriate level for tests and full reporting of outcomes
☒	☐	Estimates of effect sizes (e.g. Cohen's *d*, Pearson's *r*), indicating how they were calculated

Our web collection on statistics for biologists contains articles on many of the points above.

Software and code

Policy information about availability of computer code

Data collection	no software was used.
Data analysis	OriginLAB Origin 2020; Microsoft Excel 2019

For manuscripts utilizing custom algorithms or software that are central to the research but not yet described in published literature, software must be made available to editors/reviewers. We strongly encourage code deposition in a community repository (e.g. GitHub). See the Nature Research guidelines for submitting code & software for further information.

Data

Policy information about availability of data

All manuscripts must include a data availability statement. This statement should provide the following information, where applicable:

- Accession codes, unique identifiers, or web links for publicly available datasets
- A list of figures that have associated raw data
- A description of any restrictions on data availability

The sources of all the data used are given in the Methods section and Supplementary Information.

Field-specific reporting

Please select the one below that is the best fit for your research. If you are not sure, read the appropriate sections before making your selection.

☐ Life sciences ☐ Behavioural & social sciences ☒ Ecological, evolutionary & environmental sciences

For a reference copy of the document with all sections, see nature.com/documents/nr-reporting-summary-flat.pdf

Ecological, evolutionary & environmental sciences study design

All studies must disclose on these points even when the disclosure is negative.

Study description	In this study, we forcasted the recycling potential of anthropogenic minerals and 23 types of the capsulated materials, and their evolution from 2010 to 2050 in China. Then the meeting potential of anthropogenic mineral supply for future resource demand was examined in detail. The obtained results are validated well with the full comparison, uncertainty analysis, and sensitivity analysis.
Research sample	E-waste is one type of anthropogenic minerals. While the production, importation, and exportation of electronics were collected, the consumption was determined until 2050. Based on the Weibull lifespan equation, the generation of e-waste can be determined from 2010 to 2050. With the detailed composition of material, the evolution of material in e-waste was mapped until 2050.
Sampling strategy	In relation to the above, the error bar or shade was determined with a simple random sampling. The full data simulation is employed for Monte Carlo in uncertainty analysis.
Data collection	The data used in this study were collected from various open sources, which have been given or refereed in the Methods section and Supplementary Information.
Timing and spatial scale	The material composition of anthropogenic minerals is changing with the evolution of technology. The relevant data in this study was obtained before 2019.
Data exclusions	Based on the instructions given inside this box, this section is not applicable to this study.
Reproducibility	To ensure the reproducibility of this study, we made sure that the Method section is explicit and specific. We also provide the flow diagram of research route (Supplementary Figures 5 and 7).
Randomization	This is not applicable to our study. It is because we did not perform this type of analysis.
Blinding	This is not applicable to our study. It is because we did not perform this type of analysis.

Did the study involve field work? ☐ Yes ☒ No

Reporting for specific materials, systems and methods

We require information from authors about some types of materials, experimental systems and methods used in many studies. Here, indicate whether each material, system or method listed is relevant to your study. If you are not sure if a list item applies to your research, read the appropriate section before selecting a response.

Materials & experimental systems

n/a	Involved in the study
☒	☐ Antibodies
☒	☐ Eukaryotic cell lines
☒	☐ Palaeontology
☒	☐ Animals and other organisms
☒	☐ Human research participants
☒	☐ Clinical data

Methods

n/a	Involved in the study
☒	☐ ChIP-seq
☒	☐ Flow cytometry
☒	☐ MRI-based neuroimaging
